# A direct spino-cortical circuit bypassing the thalamus modulates nociception

**DOI:** 10.1038/s41422-023-00832-0

**Published:** 2023-06-13

**Authors:** Bing Cai, Dan Wu, Hong Xie, Yan Chen, Huadong Wang, Sen Jin, Yuran Song, Anan Li, Shiqi Huang, Sashuang Wang, Yingjin Lu, Lan Bao, Fuqiang Xu, Hui Gong, Changlin Li, Xu Zhang

**Affiliations:** 1 Guangdong Institute of Intelligence Science and Technology, Hengqin, Zhuhai, Guangdong China; 2 SIMR Joint Lab of Drug Innovation, Shanghai Advanced Research Institute, Chinese Academy of Sciences (CAS); Xuhui Central Hospital, Shanghai, China; 3 Research Unit of Pain Medicine, Chinese Academy of Medical Sciences, Hengqin, Zhuhai, Guangdong China; 4 Institute of Neuroscience and State Key Laboratory of Neuroscience, Center for Excellence in Brain Science and Intelligence Technology, CAS, Shanghai, China; 5 Shenzhen Key Laboratory of Viral Vectors for Biomedicine, Brain Cognition and Brain Disease Institute, Shenzhen Institute of Advanced Technology, CAS, Shenzhen, Guangdong China; 6 HUST-Suzhou Institute for Brainsmatics, JITRI Institute for Brainsmatics, Suzhou, Jiangsu China; 7 School of Life Science and Technology, ShanghaiTech University, Shanghai, China; 8 Department of Pain Medicine and Shenzhen Municipal Key Laboratory for Pain Medicine, Shenzhen Nanshan People’s Hospital, Shenzhen, Guangdong China; 9 State Key Laboratory of Cell Biology, Shanghai Institute of Biochemistry and Cell Biology, Center for Excellence in Molecular Cell Science, CAS, Shanghai, China; 10 Present Address: Institute of Photonic Chips; School of Medical Instrument and Food Engineering, University of Shanghai for Science and Technology, Shanghai, China

**Keywords:** Cell biology, Biological techniques

## Abstract

Nociceptive signals are usually transmitted to layer 4 neurons in somatosensory cortex via the spinothalamic-thalamocortical pathway. The layer 5 corticospinal neurons in sensorimotor cortex are reported to receive the output of neurons in superficial layers; and their descending axons innervate the spinal cord to regulate basic sensorimotor functions. Here, we show that a subset of layer 5 neurons receives spinal inputs through a direct spino-cortical circuit bypassing the thalamus, and thus define these neurons as spino-cortical recipient neurons (SCRNs). Morphological studies revealed that the branches from spinal ascending axons formed a kind of disciform structure with the descending axons from SCRNs in the basilar pontine nucleus (BPN). Electron microscopy and calcium imaging further confirmed that the axon terminals from spinal ascending neurons and SCRNs made functional synaptic contacts in the BPN, linking the ascending sensory pathway to the descending motor control pathway. Furthermore, behavioral tests indicated that the spino-cortical connection in the BPN was involved in nociceptive responses. In vivo calcium imaging showed that SCRNs responded to peripheral noxious stimuli faster than neighboring layer 4 cortical neurons in awake mice. Manipulating activities of SCRNs could modulate nociceptive behaviors. Therefore, this direct spino-cortical circuit represents a noncanonical pathway, allowing a fast sensory-motor transition of the brain in response to noxious stimuli.

## Introduction

Nociceptive signals generated in the dorsal root ganglion (DRG) neurons are known to be transmitted through the ascending axons of spinal projection neurons (SPNs) and then following thalamocortical afferents to the neurons in layer 4 of primary somatosensory cortex.^1^ The pyramidal neurons in cortical layer 5 receive the output signals of superficial neurons, and project the descending axons along the corticospinal tract (CST) to the spinal cord to regulate basic sensorimotor functions.^2^ Interestingly, some early histochemical studies on the spinal injury-induced degeneration of ascending axons in human CST and sensorimotor cortex implied a possibility of a direct spino-cortical tract.^3–8^ However, the existence of such a tract was argued by several discrepant findings.^7,8^ Further study was not able to label spinal neurons by the retrograde tracer applied into the sensorimotor cortex.^9^ Therefore, it remains unknown whether the spinal ascending axons directly regulate the activity of cortical neurons.

The basilar pontine nucleus (BPN) is located in the ventral pons, surrounding the descending tracts originated from the cerebral cortex.^10^ Neurons in the BPN are predominantly innervated by layer 5 neurons of multiple cerebral cortex regions in a spatially organized manner.^11,12^ Additionally, BPN also receives projections from several subcortical regions including spinal cord, superior colliculus and red nucleus.^13–15^ The main output axons of BPN neurons form a source of mossy fibers that convey information to the granule cells in the cerebellum.^10,12^ Therefore, BPN is considered as a precerebellar relay nucleus that transmits motor information from cerebral cortex to the cerebellum.^16^ However, little is known about the role of BPN in the sensory transmission besides motor regulation.

Here, we found that a subset of layer 5 corticospinal neurons (CSNs), namely spino-cortical recipient neurons (SCRNs), in sensorimotor cortex received direct inputs from SPNs in the deep laminae of dorsal horn. The SPNs and SCRNs made the anatomic and functional synaptic contacts in the dorsalmedial part of the BPN to form a direct spino-cortical circuit. We further detected that the spino-cortical connection in the BPN was involved in nociceptive responses. Importantly, SCRNs responded to peripheral nociceptive electrical stimuli faster than neighboring layer 4 neurons in awake mice. Moreover, SCRNs participated in the regulation of nociceptive responses and chronic pain. Thus, our study uncovers a direct spino-cortical circuit and its important role in nociceptive responses, providing new insight into understanding how the brain processes sensory information.

## Results

### Spinal projection neurons have a direct connection with layer 5 neurons in sensorimotor cortex

**Fig. 1:**
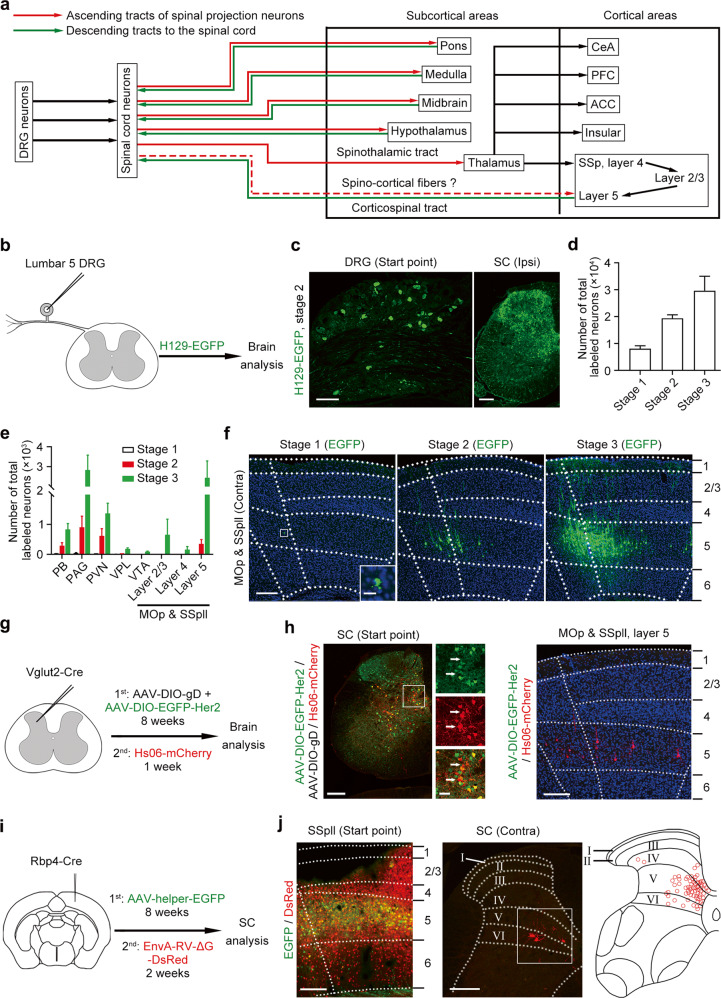
Identification of a direct projection from lumbar spinal cord to cerebral cortex. **a** Schematic diagram summarizing the ascending and descending pathways in the somatosensory transmission. **b** Schematic diagram showing the injection of H129-EGFP into left lumbar 5 DRG of wild-type mice. **c** Representative images showing EGFP-labeled neurons in the DRG and ipsilateral (Ipsi) spinal cord (SC) after H129-EGFP injection. *n* = 19. Scale bars, 200 μm. **d** Statistical result showing the 3 propagation stages of HSV-labeled brain samples (*n* = 8 for stage 1, *n* = 5 for stage 2, *n* = 6 for stage 3). **e** Statistical result showing the number of EGFP-labeled neurons in the PB, PAG, PVN, VPL, VTA, MOp and SSpll in 3 propagation stages (*n* = 8 for stage 1, *n* = 5 for stage 2, *n* = 6 for stage 3). **f** Representative images showing EGFP-labeled neurons in the contralateral (Contra) MOp and SSpll (*n* = 8 for stage 1, *n* = 5 for stage 2, *n* = 6 for stage 3). Scale bars, 200 μm (left) and 20 μm (right). **g** Schematic diagram showing HSV-based anterogradely trans-monosynaptic tracing from lumbar spinal cord. **h** Left, representative image showing the start neurons expressing EGFP and mCherry by HSV tracing in the lumbar spinal cord (*n* = 4). Scale bars, 200 μm (left) and 50 μm (right). White arrows indicate anterogradely mono-transsynaptic labeled neurons in the spinal cord. Right, mCherry-labeled other than EGFP-labeled neurons in the layer 5 of SSpll and MOp of Vglut2-Cre mice (*n* = 4). Scale bar, 200 μm. **i** Schematic diagram showing rabies virus-based retrogradely trans-monosynaptic tracing from SSpll. **j** Left, an image showing the start neurons in cortical layer 5 of Rbp4-Cre mice. Middle, DsRed-labeled neurons are located in the lamina V–VI of contralateral spinal cord. Scale bars, 200 μm. Right, the distribution of 72 DsRed-labeled projection neurons in the lumbar spinal cord (*n* = 3).

To explore the somatosensory neural network in the brain arising from DRG neurons (Fig. 1a), we performed the unilateral injection of anterogradely trans-polysynaptic type 1 herpes simplex virus (HSV-1) H129 strain expressing enhanced green fluorescent protein (H129-EGFP) into the lumbar 5 DRG (Fig. 1b). The immunosuppressor bortezomib was applied to facilitate HSV infection according to a previous report.^17^ We observed EGFP-labeled neurons in the DRG and ipsilateral spinal cord after viral injection, showing successful infection and anterogradely tans-polysynaptic spreading of H129-EGFP (Fig. 1c). We examined the expression of apoptosis marker caspase3 in the DRG and spinal cord to detect dying neurons (Supplementary information, Fig. S1a, b). The samples with no expression of caspase3 were used for further study.

According to the total number of EGFP-labeled neurons in the brain, we divided brain samples into 3 propagation stages, the average number of labeled neurons in each stage was 8069 ± 1076, 19,368 ± 1320 and 29,664 ± 5349, respectively (Fig. 1d). Previous studies reported that the parabrachial nucleus (PB), periaqueductal gray (PAG), paraventricular nucleus (PVN) and ventral posterolateral thalamic nucleus (VPL) are the major targets of SPNs.^18^ We observed EGFP-labeled neurons in the above-mentioned brain regions and found that the numbers of labeled neurons were positively correlated with propagation stages (Fig. 1e; Supplementary information, Fig. S1c). Importantly, neurons in brain nuclei that did not receive direct projection from the spinal cord, such as the ventral tegmental area (VTA),^19^ could be steadily labeled by EGFP in propagation stage 3 (Fig. 1e), suggesting that H129-EGFP could spread to the third-order nuclei of SPNs in the somatosensory neural network. Thus, the data attested to the reliability of this approach for labeling the somatosensory neural network in the brain arising from DRG.

Unexpectedly, we observed sporadic EGFP-expressing layer 5 neurons in the contralateral MOp and SSpll while no EGFP-labeled neurons could be detected in the contralateral VPL in propagation stage 1 (Fig. 1f; Supplementary information, Fig. S1c). We detected a few EGFP-labeled neurons in the VPL and a population of EGFP-positive cortical layer 5 neurons in propagation stage 2 (Fig. 1f; Supplementary information, Fig. S1c). However, no EGFP-expressing cortical layer 4 neurons could be observed in stage 2 (Fig. 1f), implying that EGFP-labeled neurons in cortical layer 5 might receive direct inputs from spinal cord as well. In propagation stage 3, we observed the increased number of EGFP-positive neurons in VPL and cortical layer 5 (Fig. 1f; Supplementary information, Fig. S1c), and EGFP-expressing neurons could be detected in cortical layer 4 (Fig. 1f), suggesting that EGFP-labeled VPL neurons and cortical layer 5 neurons were second-order neurons while EGFP-expressing cortical layer 4 neurons were third-order neurons of SPNs. Therefore, there is a possibility that a population of layer 5 neurons in MOp and SSpll might receive direct inputs from SPNs bypassing the thalamus.

To confirm this direct spino-cortical connection, we adopted the anterogradely trans-monosynaptic tracing by using genetically modified H129 mutant virus. The new H129 recombinant, named Hs06, was recently reported to enter defined neurons due to the specific recognition of truncated human epidermal growth factor receptor 2 (Her2CT9).^20^ Moreover, Hs06 could complete the packaging of the progenies with compensatory expression of codon-optimized glycoprotein D (cmgD) in the defined neurons, and then infected the downstream neurons trans-monosynaptically. To anterogradely trace the recipients of Vglut2-positive spinal projection neurons, we injected Hs06 labeled with the red fluorescent protein (Hs06-mCherry) following the expression of adeno-associated virus (AAV) helpers AAV2/9-hSyn-DIO-EGFP-T2A-Her2CT9 (AAV-DIO-EGFP-Her2) and AAV2/9-UL26.5p-DIO-cmgD (AAV-DIO-gD) in the lumbar spinal cord of Vglut2-Cre mice (Fig. 1g). We observed both EGFP/mCherry-positive start neurons and mCherry-positive second-order neurons in the spinal cord (Fig. 1h). We found mCherry other than EGFP expression in a population of layer 5 neurons in the SSpll and MOp (Fig. 1h; Supplementary information, Fig. S2a, b), indicating that Hs06-mCherry in the infected SPNs could be anterogradely transported into the cortical layer 5 neurons. As a control, Hs06 could not infect spinal neurons without expressing Her2 (Supplementary information, Fig. S2c), showing the specific infection phenotype of Hs06 virus. Although Hs06 could infect Her2-expressing spinal neurons, they could not complete the packaging of the progenies and initiate anterograde trans-monosynaptic spreading to downstream neurons in the spinal cord and cortex without the compensatory expression of HSV cmgD (Supplementary information, Fig. S2d, e). Therefore, the mCherry-labeled cortical layer 5 neurons are anterogradely labeled, but not by the terminal absorption of AAVs or HSV. Thus, we named these layer 5 neurons as SCRNs.

At the same time, we injected the EnvA-pseudotyped glycoprotein (G)-deleted rabies virus (EnvA-RV-ΔG-DsRed)^21^ after expression of AAV2/9-DIO-RVG-TVA-EGFP for 2 months in the SSpll of Rbp4-Cre mice, in which Cre was expressed in cortical layer 5 neurons, to retrogradely map the presynaptic inputs to layer 5 neurons in MOp and SSpll (Fig. 1i). Besides the DsRed-labeled neurons in the contralateral SSpll and MOp, the ipsilateral secondary somatosensory cortex and VPL of the brain were observed (Supplementary information, Fig. S2f) as previously reported^22^; and we found a group of DsRed-labeled SPNs located frequently in the medial portion of contralateral lamina IV to VI of lumbar spinal cord (Fig. 1j). To rule out the possibility that retrograde transport of helper AAV might label thalamic Cre-positive neurons as start neurons, we counted the EGFP-positive and DsRed-positive neurons in the thalamus. The statistical result showed that only ~0.2% of DsRed-labeled thalamic neurons expressed EGFP (Supplementary information, Fig. S2f). Taken together, these data indicate that a direct connection exists between SPNs and SCRNs without the relay in the thalamus.

### Spinal ascending axons directly contact cortical descending axons via a special structure of spino-cortical connecting disc in the BPN

**Fig. 2:**
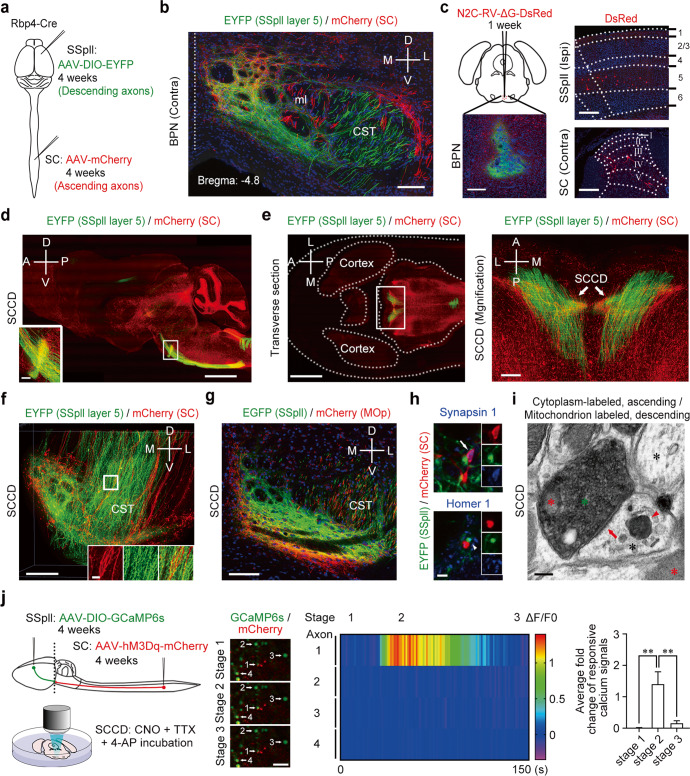
Synaptic contacts between axons from SPNs and SCRNs in the BPN. **a** Schematic diagram showing the injections of AAV viral tracers into SSpll and spinal cord of Rbp4-Cre mice. **b** EYFP-labeled fibers from SCRNs and mCherry-labeled fibers from SPNs in the BPN (*n* = 3). Scale bar, 100 μm. M, medial. L, lateral. The vertically dotted line indicates the midline of the coronal section of the brain. **c** Schematic diagram (top left) and the image (bottom left) showing the injection of retrograde rabies virus into the BPN. DsRed-labeled neurons in SSpll layer 5 neurons (top right) and deep laminae of contralateral SPNs (bottom right) (*n* = 3). Scale bars, 200 μm. **d** A representative image showing the connection between SPNs and SCRNs in the sagittal view of SCCD by fMOST (*n* = 4). Scale bars, 200 μm (left) and 1 mm (right). **e** Left, the holistic morphology and location of SCCD. Scale bar, 2 mm. Right, a magnification of CST and SCCD by rotating 90° clockwise of boxed area showing the distribution of both axon fibers from SPNs and SCRNs at the SCCD. Scale bar, 200 μm. **f** The 3D reconstruction by fMOST showing the axon fibers from SPNs (mCherry) and SCRNs (EYFP) across the CST at the SCCD (*n* = 4). Scale bars, 200 μm (left) and 50 μm (right). **g** A representative image showing axon fibers of SSpll (EYFP) and MOp (mCherry) neurons organized spatially at the SCCD (*n* = 3). Scale bar, 100 μm. **h** Top, the mCherry-labeled axon terminal from SPNs is colocalized with a presynaptic marker synapsin 1 (arrow) at the SCCD. Bottom, the EYFP-labeled axon terminal from SCRNs is co-localized with a postsynaptic marker homer 1 (arrowhead) at the SCCD (*n* = 4). Scale bar, 2 μm. **i** A representative image of electron microscopy showing the synapse (red arrow), the labeled axon terminals of SPNs (cytosol, green asterisk) and SCRNs (mitochondrion, red arrowhead) at the SCCD (*n* = 3). The black asterisks indicate unlabeled cytoplasm and red asterisks indicate unlabeled mitochondria. Scale bar, 500 nm. **j** Left, schematic diagram showing the injections of AAVs expressing GCaMP6s and hM3Dq into SSpll and lumbar spinal cord of Rbp4-Cre mice, respectively. Middle, representative images and heatmap showing increased calcium signals of indicated cortical descending axon terminal with chemogenetic activation of spinal ascending axons at the SCCD. Scale bar, 5 μm. Right, statistical result showing the change of calcium activities of responsive axons (*n* = 16 from 10 mice). **P < 0.01, two-tailed paired *t*-test. Da*t*a shown are mean ± SEM.

Given that SPNs send a direct projection to SCRNs, where is this connection located in the brain? We firstly excluded the possibility of these connected axon terminals in the somatosensory cortex. We injected non-transsynaptic AAV2/9-hSyn-EGFP unilaterally into the lumbar spinal cord to trace the spinal ascending axons and observed the labeled axons in the PB, VPL and LHA, but not in the somatosensory cortex (Supplementary information Fig. S2g). Then, we synchronously performed the anterogradely and retrogradely non-transsynaptic tracing technique to find the connected region between ascending axons from SPNs and descending axons from SCRNs. The AAV2/9-hSyn-mCherry (AAV-mCherry) was injected unilaterally into the lumbar spinal cord to trace the ascending axons from SPNs, and the AAV2/9-EF1a-DIO-EYFP (AAV-DIO-EYFP) was injected into the contralateral SSpll to trace the descending axons from layer 5 neurons of SSpll in Rbp4-Cre mice (Fig. 2a; Supplementary information, Fig. S3a). Immunohistochemical staining of serial sections in the brain showed that the mCherry-labeled ascending axons from SPNs and the EYFP-labeled descending axons from layer 5 neurons of SSpll exhibited distinct distributions, but did not display co-innervation in most brain regions such as VPL, LAT, and nucleus tractus solitarii (NTS) (Supplementary information, Fig. S3b). Importantly, we did identify their co-innervation in the BPN close to the midline of ventral pons (Bregma: –4.8, Fig. 2b). For long-term study on the SCRN functions, we also tried to use anterogradely trans-monosynaptic AAV2/1-hSyn-Cre (AAV2/1-Cre), which has less toxicity than HSV, to trace the neurons. To test whether the SCRNs could be labeled by both anterogradely trans-monosynaptic HSV (HS06-mCherry) and AAV (AAV2/1-Cre), we unilaterally injected Hs06-mCherry after the expression of AAV2/1-Cre, AAV-DIO-EGFP-Her2 and AAV-DIO-gD in the lumbar spinal cord, and the AAV2/9-CAG-DIO-EBFP into the contralateral SSpll (Supplementary information, Fig. S3c). Under the circumstance, the mCherry-labeled neurons were infected by Hs06 and the expression of EBFP could be driven by anterogradely trans-monosynaptic AAV2/1-Cre in cortical layer 5 neurons. Immunohistochemical staining showed that ~78% mCherry-labeled cortical layer 5 neurons also express EBFP while ~82% EBFP-positive cortical layer 5 neurons also express mCherry (Supplementary information, Fig. S3c), suggesting that SCRNs are labeled by both Hs06 and AAV2/1-Cre. Then we injected a mixture of AAV-mCherry and AAV2/1-Cre unilaterally into the lumbar spinal cord to label SPNs with mCherry, followed by the injection of AAV-DIO-EYFP into the contralateral side of SSpll to allow the expression of EYFP in SCRNs driven by AAV2/1-Cre (Supplementary information, Fig. S3d). Immunohistochemical staining further showed the distribution of mCherry-labeled ascending axons from SPNs and the EYFP-labeled descending axons from SCRNs in the BPN (Supplementary information, Fig. S3e), suggesting that BPN receives projections from both SPNs and SCRNs. Moreover, we injected the retrogradely non-transsynaptic engineered rabies virus (N2C-RV-ΔG-DsRed)^23^ with cholera toxin subunit B488 (CTB488) into the BPN of wild-type mice to trace the innervating neurons (Fig. 2c). We observed DsRed-labeled neurons in both the layer 5 of ipsilateral sensorimotor cortex and the contralateral deep laminae of spinal cord (Fig. 2c). Additionally, the injection of AAV2/retro-Cre into the BPN of Rosa26-tdTomato (Ai9) reporter mice also displayed the tdTomato-expressing neurons in ipsilateral sensorimotor cortex and contralateral spinal cord (Supplementary information, Fig. S3f). Therefore, the BPN in ventral pons is a critical region establishing a direct contact between the ascending axons from SPNs and the descending axons from SCRNs.

We further used the fluorescence micro-optical sectioning tomography system (fMOST)^24^ to precisely map both the spinal ascending axons from SPNs and the descending axons from cortical layer 5 SCRNs. We observed the CST cross in the ventral pons, suggesting that the descending axons from SCRNs were a part of CST (Fig. 2d). The transverse section showed the cross as a hump of CST (Fig. 2e). Interestingly, fMOST 3D reconstruction showed that the axon fibers from SPNs and SCRNs formed a disciform structure derived from the bundles of spinal ascending axons and cortical descending axons in the BPN (Fig. 2f; Supplementary information, Fig. S3g and Video S1). We named this specific structure as the spino-cortical connecting disc (SCCD), which is located in the dorsalmedial part of the BPN. The major axis and minor axis of SCCD were ~600 μm and ~350 μm, respectively (Fig. 2f; Supplementary information, Fig. S3g). The thickness of SCCD was ~50 μm. To further illuminate the spatial distribution of descending axon fibers from sensorimotor cortex, we injected AAV2/9-hSyn-mCherry and AAV2/9-hSyn-EGFP into the adjacent MOp and SSpll, respectively (Supplementary information, Fig. S3h). The descending axons from layer 5 neurons in both sensory and motor cortices contributed to the axonal network at the SCCD in a spatially organized manner. Immunohistochemical staining showed that EGFP-labeled descending axons from SSpll projected to the medial part of SCCD while mCherry-labeled descending axons from MOp innervated the lateral part of SCCD (Fig. 2g). In addition, the enlarged confocal image and the rotated image from fMOST 3D reconstruction both clearly showed that the ascending and descending axons labeled in the bundles adjoining the BPN were separated (Fig. 2b, f; Supplementary information, Fig. S3g), excluding the possibility of leaky expression of fluorescent proteins. These results combined suggest that spinal ascending axons directly contact cortical descending axons in the BPN with a specific structure of SCCD.

### Spinal ascending axons form functional synapses with cortical descending axons at the SCCD of BPN

Given that specific contacts in the BPN existed between spinal ascending axons and cortical descending axons, we examined the localization of synaptic markers at the SCCD. Immunohistochemical staining showed that the presynaptic synapsin 1-positive puncta in the spinal ascending axon terminals were in contact with the postsynaptic homer1-positive puncta in the cortical descending axon terminals at the SCCD (Fig. 2h). Moreover, using multiplexed peroxidase-based electron microscopy,^25^ we examined whether the ascending axons from SPNs could make real synaptic contacts with the descending axons from SCRNs at the SCCD. We synchronously injected the AAV2/9-EF1a-dAPEX2 (AAV-dAPEX2) unilaterally into the lumbar spinal cord to express peroxidase dAPEX2 in the cytosol of the ascending axons from SPNs, and the AAV2/9-EF1a-DIO-COX4-dAPEX2 (AAV-DIO-COX4-dAPEX2) into the contralateral SSpll to express peroxidase dAPEX2 in the mitochondria of descending axons from SCRNs in Rbp4-Cre mice (Supplementary information, Fig. S4a). The mitochondrial labeling of dAPEX2 represented the cortical descending axons, while the cytosol labeling of dAPEX2 marked the spinal ascending axons (Supplementary information, Fig. S4b). Notably, the presynaptic buttons of spinal ascending axons often formed synapses with the postsynaptic buttons of cortical descending axons (Fig. 2i; Supplementary information, Fig. S4b–d). Interestingly, we also observed that the mitochondria-labeled descending axons from SCRNs contained synaptic vesicles (Supplementary information, Fig. S4b), implying that SCRNs control BPN neurons by descending axons. Thus, the spinal ascending axons from SPNs indeed make synaptic contacts with the cortical descending axons from SCRNs at the SCCD.

To further examine the functional connectivity between the spinal ascending terminals from SPNs and the cortical descending terminals from SCRNs, we adopted in vitro calcium imaging on the brain slices in combination with a chemogenetic method of designer receptor (hM3Dq) exclusively activated by designer drug (DREADD). We injected AAV2/9-EF1a-hM3Dq-mCherry (AAV-hM3Dq-mCherry) unilaterally into the lumbar spinal cord, and AAV2/9-hSyn-DIO-GCaMP6s (AAV-DIO-GCaMP6s), a genetically encoded fluorescent calcium indicator, into the contralateral SSpll of Rbp4-Cre mice (Fig. 2j). One month after viral injection, 400-μm thick acute brain slices were prepared with the vibratome. The slice containing mCherry-positive spinal ascending axons and GCaMP6s-positive cortical descending axons in SCCD was examined under a fluorescence microscope and selected for in vitro calcium imaging. Then the slice was embedded in the low-melting-point agarose and GCaMP6s-positive axons were further confirmed by 25× water immersion lens of Olympus FVMPE-RS microscope. The calcium activity of cortical descending terminals from SCRNs at the SCCD was monitored during the chemogenetic activation of spinal ascending terminals containing hM3Dq by incubating oxygenated artificial cerebrospinal fluid (ACSF) containing Clozapine N-oxide (CNO, 200 μM), 1 μM tetrodotoxin (TTX) and 100 μM 4-aminopyridine (4-AP). Presynaptic hM3Dq-containing spinal ascending axons in SCCD could be activated by CNO. TTX and 4-AP were used to prevent the polysynaptic transmission during the test.^26^ Therefore, the responsive calcium signals of GCaMP6s-positive cortical descending axons observed in SCCD during the experiment would be monosynaptically activated by presynaptic hM3Dq-containing spinal ascending terminals. As expected, ~1.4-fold increase in calcium signal was induced in the descending axon terminals by chemogenetic activation of presynaptic axon terminals containing hM3Dq (Fig. 2j). These data indicate that spinal ascending axons establish a direct functional connectivity with cortical descending axons.

### Direct spino-cortical circuit links spinothalamic tract (STT) and CST

**Fig. 3:**
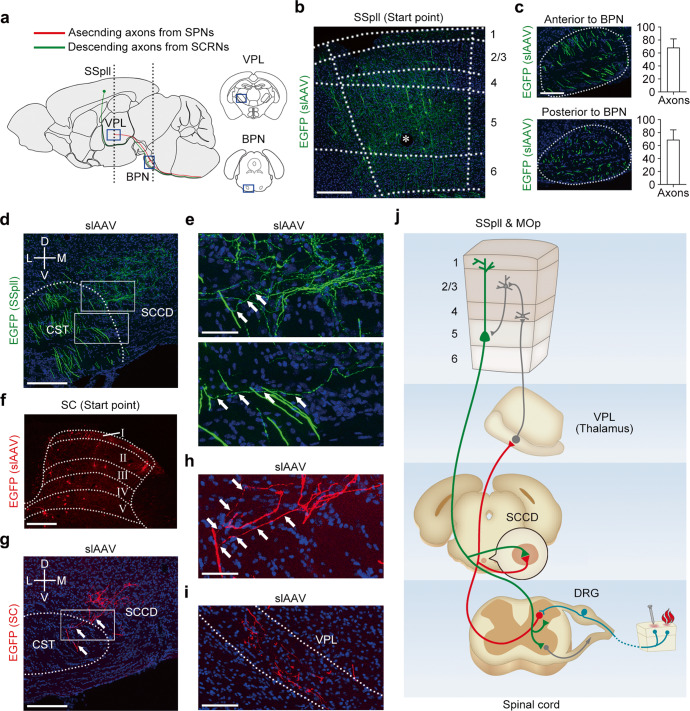
Branches of STT and CST at SCCD form spino-cortical circuit. **a** Schematic diagram showing the axon tracks from SPNs and SCRNs. **b** EGFP-labeled neurons infected by slAAV at SSpll. The asterisk indicates the injection site (*n* = 3). Scale bar, 200 μm. **c** Representative images and statistics results showing that the total number of axons in the CST is not changed before and after branching to the SCCD (*n* = 3). Scale bar, 200 μm. **d** Representative image showing that EGFP-labeled descending axons in the CST branch out (white arrows) and terminate at the SCCD (*n* = 3). Scale bar, 200 μm. **e** The enlarged images of white squares in **d** showing the branches (white arrows) of EGFP-labeled axons in the CST. Scale bar, 50 μm. **f** A representative image showing EGFP-labeled neurons (presented as red) infected by slAAV in the lumbar spinal cord (*n* = 3). Scale bar, 200 μm. **g** A representative image showing that EGFP-labeled ascending axons (presented as red) from SPNs branch out (white arrows) at the SCCD (*n* = 3). Scale bar, 200 μm. **h** The enlarged image of white square in **g** showing the branches (white arrows) of EGFP-labeled axons from SPNs. Scale bar, 50 μm. **i** Representative image showing that EGFP-labeled ascending axons from SPNs terminate at VPL (*n* = 3). Scale bar, 200 μm. **j** Proposed model for the direct spino-cortical circuit formed by synaptic connection between the branches of cortical descending axons and spinal ascending axons at the SCCD.

It was unclear whether a subset of axons from SPNs or SCRNs solely terminated at the SCCD or branched to the SCCD on the way to their destination. Then, we explored the exact anatomical circuits of SPNs and SCRNs at the SCCD by applying sparse-labeling AAV (slAAV)^27^ to label neurons and their axons clearly (Fig. 3a). We injected slAAV into the SSpll to express EGFP in a small population of neurons (Fig. 3b). The number of descending axons from SSpll layer 5 neurons in the CST anterior and posterior to the BPN did not display significant difference (Fig. 3c). Importantly, a portion of descending axons branched to and terminated at the SCCD (Fig. 3d, e), indicating that the axon fibers innervating the SCCD from SSpll layer 5 neurons are the branches of CST. Furthermore, we labeled spinal neurons via the injection of slAAV into the lumbar spinal cord (Fig. 3f). Neural tracing showed that the spinal ascending axons were located dorsally and laterally to the CST, and branched to and terminated at the SCCD (Fig. 3g, h). These spinal ascending axons finally terminated at the VPL (Fig. 3i), suggesting that the spinal ascending axons innervating the SCCD are the branches of STT that originated from SPNs in the spinal cord. Therefore, the STT and CST are bridged via the spino-cortical circuit, which may lead to a fast sensory-motor transition in response to peripheral stimuli (Fig. 3j).

### Spino-cortical connection in the BPN modulates nociceptive responses

**Fig. 4:**
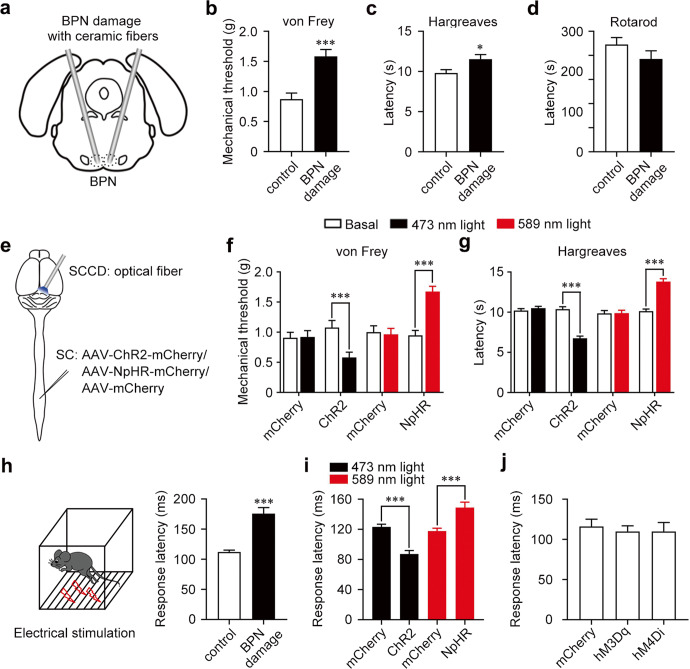
Contribution of spino-cortical connection to nociceptive responses. **a** Schematic diagram showing the bilateral damage of BPN by ceramic fibers. **b**–**d** Nociceptive mechanical threshold in von Frey test (**b**), noxious thermal latency in Hargreaves test (**c**), and rotarod test showing motor ability (**d**) after BPN damage. *P < 0.05, ***P < 0.001 vs control. *n* = 11 for control and *n* = 12 for BPN damage. **e** Schematic diagram showing the expression of optogenetic components in SPNs and the implant of optical fibers above the SCCD. **f**, **g** Nociceptive mechanical threshold in the von Frey test (**f**) and noxious thermal latency in Hargreaves test (**g**) during optogenetic manipulations at the SCCD. ***P < 0.001 vs basal. *n* = 13 for ChR2, *n* = 14 for mCherry; *n* = 14 (**f**) and *n* = 11 (**g**) for NpHR, *n* = 11 for mCherry groups. **h** Left, schematic diagram of noxious electrical foot shock. Right, the response latency of noxious electrical foot shock after BPN damage. ***P < 0.001 vs control. *n* = 11 for control and *n* = 12 for BPN damage. **i** Response latency of noxious electrical foot shock during optogenetic manipulations at the SCCD. ***P < 0.001 vs mCherry. *n* = 14 for ChR2, *n* = 10 for mCherry; *n* = 10 for NpHR, *n* = 15 for mCherry groups. **j** Response latency of noxious electrical foot shock after chemogenetic manipulation of BPN local neurons. *n* = 9 for both mCherry and hM3Dq groups, and *n* = 10 for hM4Di group. Data shown are mean ± SEM. Two-tailed unpaired *t*-test.

To explore the role of BPN in nociceptive responses, we damaged the bilateral BPNs by implanting ceramic fibers into the brain (Fig. 4a; Supplementary information, Fig. S5a). The BPN-damaged mice exhibited a significantly heightened nociceptive mechanical threshold, and increased the response latency to noxious thermal stimuli compared to control mice (Fig. 4b, c), implying that BPN modulates nociceptive responses. BPN may affect motor behaviors besides nociceptive responses as the cerebellum is the major downstream target of BPN projection neurons.^11^ We further performed behavioral tests to explore the role of BPN in motor regulation. The rotarod and beam walking tests showed that the damage of BPN did not affect the motor coordination compared to control mice (Fig. 4d; Supplementary information, Fig. S5b). At the same time, the footprint analysis showed that the BPN-damaged mice did not exhibit the defect of primary locomotor gait (Supplementary information, Fig. S5c). Thus, BPN is involved in nociceptive responses.

Since the spino-cortical circuit made direct synaptic contacts in the BPN, we further distinguished the role of spino-cortical connection from local neurons in the BPN in response to nociceptive stimulation. We injected AAV2/9-EF1a-hM3Dq-mCherry or AAV2/9-EF1a-hM4Di-mCherry into bilateral sides of the BPN to express chemogenetic components in local neurons. Chemogenetic manipulations of local neurons in the BPN with intraperitoneal injection of 1 mg/kg CNO did not show significant impact on nociceptive mechanical threshold, response latency to noxious thermal stimuli and motor coordination in mice, suggesting that local neurons in the BPN are not involved in nociceptive responses (Supplementary information, Fig. S5d–f). To further determine the function of synaptic connectivity between SPNs and SCRNs, we expressed excitatory channelrhodopsin-2-mCherry (AAV2/9-hSyn-ChR2-mCherry, AAV-ChR2-mCherry) or inhibitory halorhodopsin-mCherry (AAV2/9-hSyn-NpHR3.0-mCherry, AAV-NpHR-mCherry) in the lumbar spinal cord followed by implantation of optical fiber above mCherry-positive axon terminals in the BPN (Fig. 4e; Supplementary information, Fig. S5g). The optogenetic activation of ChR2 in spinal ascending axon terminals at the SCCD decreased the nociceptive mechanical threshold and the response latency to noxious thermal stimuli, while the optogenetic inhibition of NpHR increased nociceptive mechanical threshold (Fig. 4f, g). These data suggest that the direct spino-cortical connection in the SCCD of BPN regulates nociceptive responses.

The noxious electric foot shock (0.5 mA, 1 s) causes a fast escape behavior^28^ (Fig. 4h). We examined whether the direct spino-cortical circuit participated in the fast escape behavior induced by peripheral electric stimulation in mice. The damage of BPN significantly extended the response latency in mice (Fig. 4h), suggesting that BPN is involved in the fast escape behavior induced by electric foot shock. Importantly, optogenetic activation of ChR2 in spinal ascending axon terminals at the SCCD decreased the response latency to the noxious electric foot shock in mice, while the optogenetic inhibition of NpHR increased the response latency (Fig. 4i). However, chemogenetic manipulation of BPN local neurons did not affect the response latency (Fig. 4j). Taken together, the direct spino-cortical connection in the BPN modulates nociceptive responses, including the fast escape behavior to unexpected nociceptive stimuli.

### The SCRNs in spino-cortical circuit contribute to the rapid regulation of pain

**Fig. 5:**
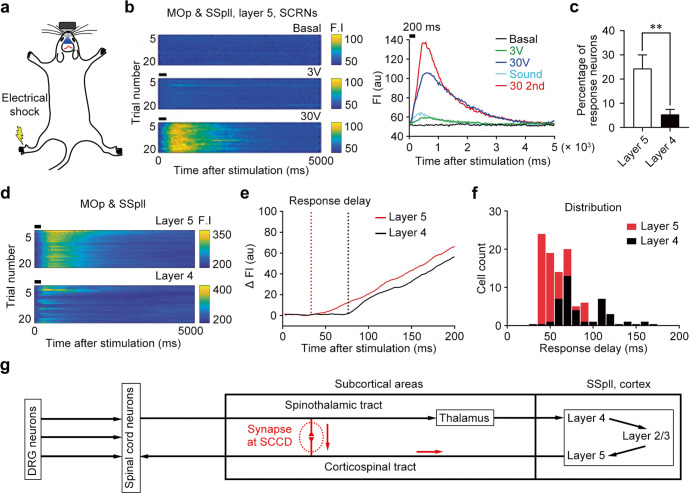
Fast response of SCRNs to peripheral noxious stimuli. **a** Schematic diagram of in vivo calcium imaging on the neurons in the SSpll during the peripheral electrical stimulation in awake mice. **b** Left, representative heatmaps illustrating the calcium activities of SCRNs associated with peripheral electrical stimuli. Right, representative calcium signals showing the specific response of SCRNs to peripheral noxious stimuli (*n* = 3). **c** Statistical results showing the percentage of responding neurons in the layer 5 and layer 4 (*n* = 7). Two-tailed unpaired *t*-test, **P < 0.01 vs layer 5. **d** Representative heatmaps showing the responses of SCRNs and layer 4 neurons to peripheral noxious stimuli (*n* = 3). **e** Representative calcium traces showing the shorter response latency of layer 5 SCRNs than layer 4 neurons (*n* = 3). **f** Response latencies of SCRNs (*n* = 92) and layer 4 neurons (*n* = 39). K–S test, ***P < 0.001. **g** Schematic diagram showing the canonical ascending spinothalamic-thalamocortical circuit, the descending cortico-spinal circuit and the direct spino-cortical circuit (red arrows) bypassing the thalamus. Data shown are mean ± SEM.

To explore the function of this direct spino-cortical circuit, we performed in vivo calcium imaging^29^ on the SSpll and MOp of mice. We monitored the neuronal activity by using the viral approach to drive the expression of GCaMP6s specifically in the layer 5 spino-projecting SCRNs or in the layer 4 Scnn1a-Cre-derived neurons (Fig. 5a; Supplementary information, Fig. S6a, b). Plantar surface electrical stimuli (30 V, 200 ms) on the hindpaw induced an intense increase of GCaMP6s signals in a subset of layer 5 SCRNs (Fig. 5b). By comparison, the low-voltage electrical stimuli (3 V, 200 ms) or the sound stimuli (70 dB, 2000 Hz, 200 ms) elicited no or only little increase of GCaMP6s signals in SCRNs (Fig. 5b). Notably, the electric stimuli (30 V, 200 ms) could induce response of ~25% SCRNs in the layer 5, more than the layer 4 neurons (~5%) (Fig. 5c; Supplementary information, Videos S2, S3), suggesting that layer 5 SCRNs is relatively more receptive to peripherally nociceptive electrical stimuli. To accurately examine the response latency, we performed the line scanning on the responsive neurons to record the calcium signals at a higher temporal resolution (Fig. 5d; Supplementary information, Fig. S6c). Importantly, the layer 5 SCRNs responded to plantar surface electrical stimuli with a speed 29% faster than layer 4 neurons (Fig. 5e, f). The response latency of both layer 5 SCRNs and layer 4 neurons showed a quantum-like distribution (Fig. 5f). The majority of layer 5 SCRNs responded with two peaks at 40 ms and 70 ms, while the layer 4 neurons mainly replied with one peak at 70 ms (Fig. 5f), indicating that the earlier response of SCRNs is unlikely due to the activation via the layer 4 neurons. Thus, the layer 5 SCRNs directly and rapidly respond to peripheral stimulation through the direct spino-cortical circuit, while the layer 4 neurons receive the information relayed by thalamic neurons (Fig. 5g).

**Fig. 6:**
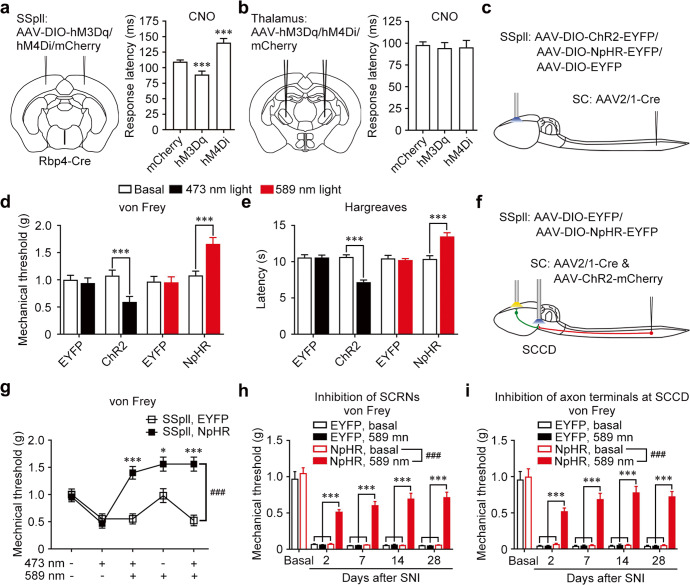
Requirement of cortical layer 5 SCRNs for noxious stimulus-evoked responses. **a** Left, schematic diagram of viral expression of hM3Dq or hM4Di in SSpll of Rbp4-Cre mice. Right, the response latency of noxious electrical foot shock after chemogenetic manipulation. ***P < 0.001 vs mCherry. *n* = 7 for mCherry group, and *n* = 8 for both hM3Dq and hM4Di groups. **b** Left, schematic diagram of viral expression of hM3Dq or hM4Di in thalamus of wild-type mice. Right, the response latency of noxious electrical foot shock after chemogenetic manipulation of activities of thalamus neurons. *n* = 8 for both mCherry and hM3Dq groups, and *n* = 7 for hM4Di group. **c** Schematic diagram showing the strategy of ChR2 or NpHR expression in SCRNs by co-injection of AAV-DIO-ChR2-EYFP or AAV-DIO-NpHR-EYFP in SSpll and AAV2/1-Cre in the lumbar spinal cord. **d** Nociceptive mechanical threshold after optogenetic activation and inhibition of SCRNs. ***P < 0.001 vs basal. *n* = 10 for ChR2, *n* = 14 for EYFP; *n* = 10 for NpHR, *n* = 13 for EYFP groups. **e** Noxious thermal latency after optogenetic activation and inhibition of SCRNs. ***P < 0.001 vs basal. *n* = 9 for ChR2, *n* = 12 for EYFP; *n* = 10 for NpHR, *n* = 13 for EYFP groups. **f** Schematic diagram showing the strategy of ChR2 expression in SPNs and NpHR expression in SCRNs by co-injection of AAV-DIO-NpHR-EYFP in SSpll and AAV2/1-Cre and AAV-ChR2-mCherry in the lumbar spinal cord. **g** Nociceptive mechanical threshold after optogenetic activation of SPN terminals at the SCCD or/and optogenetic inhibition of SCRNs. ***P < 0.001 for the 3rd and the 5th tests, *P < 0.05 for the 4th test, ###*P* < 0.001 vs SSpll, EYFP. *n* = 10 for both groups. **h** Von Frey test after SNI showing the nociceptive mechanical threshold after optogenetic inhibition of SCRNs. ***P < 0.001 vs controls. *###P* < 0.001 vs NpHR, basal. *n* = 9 for NpHR and *n* = 12 for EYFP groups. **i** Nociceptive mechanical threshold after SNI in the von Frey test during optogenetic manipulations at the SCCD. ***P < 0.001 vs controls, *###P* < 0.001 vs NpHR, basal. *n* = 10 for mCherry and *n* = 11 for NpHR groups. Data shown are mean ± SEM. Two-tailed unpaired *t*-test (**a**, **b**, **d**, **e**) or two-way ANOVA test followed by Bonferroni correction (**g**–**i**).

We further examined the role of SCRNs in the nociceptive responses of mice. The escape behavior induced by noxious electrical foot shock showed that the chemogenetic activation of layer 5 neurons by hM3Dq driven by Rbp4-Cre in the SSpll and MOp decreased the escape latency in mice. In contrast, the chemogenetic inhibition by hM4Di driven by Rbp4-Cre increased the escape latency (Fig. 6a; Supplementary information, Fig. S7a). However, the chemogenetic manipulation of neurons expressing AAV2/9-EF1a-hM3Dq-mCherry or AAV2/9-EF1a-hM4Di-mCherry in the thalamus had no significant impact on the escape behavior in wild-type mice (Fig. 6b; Supplementary information, Fig. S7b). Meanwhile, AAV2/9-EF1a-DIO-ChR2-EYFP or AAV2/9-EF1a-DIO-NpHR3.0-EYFP was injected into SSpll, and driven by the injected AAV2/1-Cre in the lumbar spinal cord to express optogenetic components including ChR2 or NpHR in SCRNs (Fig. 6c; Supplementary information, Fig. S7c). The optogenetic activation of SCRNs with ChR2 decreased the nociceptive mechanical threshold and the response latency to noxious thermal stimuli in mice, while the optogenetic inhibition with NpHR increased these nociceptive responses (Fig. 6d, e; Supplementary information, Fig. S7d). Additionally, the optogenetic manipulation of SCRN activity did not affect the motor ability (Supplementary information, Fig. S7e). At the same time, the chemogenetic manipulation of SCRN activity with hM3Dq or hM4Di obtained similar effects in mice (Supplementary information, Fig. S7f–j). Moreover, the optogenetic inhibition of layer 5 SCRNs with NpHR abolished the nociceptive mechanical hypersensitivity induced by the optogenetic activation of spinal ascending axon terminals at the SCCD with ChR2 in mice (Fig. 6f, g; Supplementary information, Fig. S7k). These data suggest that the SCRNs in direct spino-cortical circuit are involved in nociceptive responses.

Peripheral nerve injury may induce neuropathic pain, which is characterized by persistent mechanical allodynia triggered by innocuous mechanical stimuli.^30^ To detect the role of spino-cortical circuit in neuropathic pain, we used the model of spared nerve injury (SNI). As expected, mice expressing EYFP or NpHR-EYFP in SCRNs developed similar long-lasting mechanical allodynia under basal condition without optogenetic stimulation (Fig. 6h). Importantly, mice receiving optogenetic inhibition of layer 5 SCRNs expressing NpHR-EYFP markedly relieved SNI-induced mechanical allodynia compared to their basal condition or to mice expressing EYFP in layer 5 SCRNs (Fig. 6h). Meanwhile, the chemogenetic inhibition of SCRN activity with hM4Di obtained the similar effects (Supplementary information, Fig. S7l). Furthermore, mice receiving optogenetic inhibition of the ascending axon terminals from SPNs at the SCCD expressing NpHR-mCherry largely attenuated the SNI-induced mechanical allodynia compared to their basal condition or to mice expressing mCherry in SPNs (Fig. 6i). Taken together, these data indicate that the direct spino-cortical circuit consisting of SPNs, the SCCD in BPN, and SCRNs participates in pain regulation.

## Discussion

It has been generally accepted that the somatosensory transmission from the spinal cord to the somatosensory cortex relies on the STT and thalamocortical pathway relayed in the thalamus.^31^ In the present study, we identified a direct spino-cortical circuit composed of SPNs and SCRNs. The spinal ascending axons from SPNs could form direct synapses with the cortical descending axons from SCRNs at the SCCD in BPN, facilitating the fast reaction of SCRNs to peripheral stimuli. In fact, canonical STT from SPNs sends the branches to contact the branches of the CST from SCRNs at the SCCD. This direct spino-cortical circuit bypasses the relay in the thalamus, and represents a noncanonical pathway for the neurotransmission of somatosensory signals. In this regard, the nociceptive inputs from SPNs could be separately transmitted and processed in two distinct ascending neural circuits in the brain.

### A direct spino-cortical circuit for somatosensory transmission

In the present study, several recently-developed methods of viral tracing provided efficient tools for uncovering this direct spino-cortical circuit. The tracing data of somatosensory neural network arising from lumbar DRG neurons gave a hint of this circuit. The results showed that neurons in the VPL and cortical layer 5 were labeled by EGFP before cortical layer 4 neurons, which received somatosensory information from VPL neurons. Furthermore, the neural tracing by newly-developed anterogradely trans-monosynaptic virus Hs06 further attested to the existence of a direct spino-cortical connection. The successful entry and starting trans-synaptic spreading of Hs06 in defined neurons were strictly dependent on the compensatory expression of Her2CT9 and cmgD, respectively. Therefore, the possibility that labeling of layer 5 neurons in SSpll and MOp by HSV was due to the terminal absorption of helper AAVs and Hs06 could be ruled out. Meanwhile, the neural tracing by retrogradely trans-monosynaptic virus EnvA-RV-ΔG-DsRed was also applied to trace from layer 5 somatosensory cortex to SPNs. It could be suspected that Hs06 and the helper AAVs might be retrogradely transported to some vglut2-positive supra-spinal neurons with descending axons to the spinal cord, and then the progenies of Hs06 might be packaged and anterogradely transported to cortical layer 5 neurons. However, combined with all other data collected with multiple neural tracing techniques including the retrogradely trans-monosynaptic rabies virus labeling, the Hs06 expression in cortical layer 5 neurons is unlikely due to such a possibility. Moreover, 3D reconstruction by fMOST showed that the axons from SPNs and SCRNs formed a specific disciform structure named as SCCD in the BPN. The multiplexed peroxidase-based electron microscopy gave a final support that ascending terminals of SPNs made real synaptic contacts with descending terminals of SCRNs at the SCCD. The ectopic action potentials and axonal backfiring could be frequently recorded in axon terminals innervating in a restricted brain region.^32^ Both in vitro and in vivo calcium imaging showed that layer 5 SCRNs received direct inputs from axon terminals of SPNs at the SCCD and responded to peripheral nociceptive stimuli faster than cortical layer 4 neurons. Combining these morphological and functional evidences, a direct spino-cortical circuit is well established.

### SCRNs rapidly participate in nociceptive responses

Several neural circuits have been found to participate in the central regulation of nociceptive responses.^1,33,34^ The canonical spino-thalamocortical circuit consists of SPNs, thalamic neurons, cortical layer 4 neurons, layer 2/3 neurons and CSNs. However, the newly-identified spino-cortical circuit is only composed of SPNs and SCRNs, which may be the shortest circuit linking the ascending sensory tract to the descending motor control CST for the fast movement regulation in response to peripheral noxious stimuli. The spinothalamic tract may contribute to both the sensory perception and the refined movement regulation. Given that the peripheral stimulation-induced fast response of SCRNs was recorded in cortical layer 5, the action potentials generated at their axon terminals at the SCCD could be backpropagated to their cell bodies.^32^ It is possible that the ectopic action potentials generated at the axon terminals of SCRNs in the SCCD might also propagate to the axon branches in spinal cord. The interaction between canonical and ectopic spike propagations is not well understood,^32^ and the somatic hyperpolarization could reduce the firing probability of ectopic action potential.^35^ We found that inhibiting the somatic activity led to analgesic effects on the nociceptive mechanical responses induced by activating the axon terminals of SCRNs in the SCCD, suggesting that the signals generated at the soma and axon initial segments of SCRNs are required for regulating the mechanical nociception.

In vivo calcium imaging in awake mice showed that a part of SCRNs received somatosensory inputs through the direct spino-cortical circuit, while the remaining CSNs might obtain information through the spinothalamic-thalamocortical projection. These two subpopulations of CSNs are proposed to have distinct functions, responsible for pain-induced fast escaping reaction and refined movement regulation, respectively. In addition to the ascending pathways, the descending pathways derived from sensorimotor cortex are also involved in the regulation of nociceptive responses. A recent study reported that ablation of CST or CSNs affect light touch other than nociceptive responses under physiological condition.^36^ The cortical layer 5 neurons including pyramidal tract neurons exhibit molecular and projection diversities, suggesting their functional complexity.^37,38^ The effect of CSNs on light touch may be caused by ablation of multiple neuron types.

The descending pathways from MOp regulate mechanical allodynia and negative emotional valence after peripheral nerve injury in a layer-specific manner.^39^ The layer 5 MOp projections to zona incerta and PAG inhibit neuropathic mechanical allodynia. However, the tracing and single-cell RNA sequencing studies showed that distinct neuron types in MOp layer 5 send descending axons to zona incerta, PAG and spinal cord, respectively.^40,41^ Taken together, multiple neuron types in different layers in sensorimotor cortex regulate pain behaviors. Since inhibiting the activity of SCRNs at their cell bodies in the cortical layer 5 or the ascending axon terminals of SPNs at the SCCD could reduce both acute nociceptive responses and persistent pain induced by peripheral nerve injury, the SCCD containing the synapses of spino-cortical circuit plays an important role in pain modulation. Thus, inhibiting the activity of SCRNs and the direct spino-cortical circuit could be a potential approach to reduce neuropathic pain.

### BNP as a subcortical center for sensorimotor regulation

Nociceptive stimuli induce not only the motor reflex but also alert responses of whole body, leading to the transition from the sensory sensation to multiple regulatory systems in the brain. It is known that BPN receives massive projections from multiple cortical regions and subcortical areas, including the spinal cord, visual cortex and sensorimotor cortex.^12^ The newly-identified SCCD in the BPN could act as a direct connective region between the sensory cortex and the subcortical regions that processes the sensory information and/or regulates the motor responses. As a part of the ascending reticular activating system, BPN neurons may contribute to the arousal, alertness, and accelerated heartbeat induced by peripheral noxious stimuli. It has been shown that BPN receives massive convergent descending projections from multiple cortical regions and the cerebellum is a primary output target of BPN neurons.^11^ A recent study revealed that the photoinhibition of BPN neurons during a movement-planning task could decrease the correlation between the premotor cortical layer 5 neurons and the cerebellar granule cells.^42^ Our present results together with previous evidences suggest that BPN neurons receive inputs from both spinal ascending and cortical descending axon terminals. The direct synaptic contacts between the ascending axons from SPNs and the descending axons from SCRNs at the SCCD provide the structural and functional basis for the notion that BPN may serve as a subcortical center for rapidly integrating sensory and motor reactions in response to noxious stimuli.

## Materials and methods

### Animals and surgical procedures

All procedures were performed according to the guidelines of the Committee for Research and Ethical Issues of the International Association for the Study of Pain, and were approved by the Committee of Use of Laboratory Animals and Common facility, Center for Excellence in Brain Science and Intelligence Technology, CAS, and Guangdong Institute of Intelligence Science and Technology. C57BL/6J mice were purchased from Shanghai Laboratory Animal Center, CAS (Shanghai, China). Vglut2-Cre (JAX016963), Ai9 (JAX007909) and Scnn1a-Cre (JAX009613) mice were initially acquired from the Jackson Laboratory and on C57BL/6 genetic background. Rbp4-Cre (031125-UCD) mice were initially acquired from the mutant mouse resource and research center (MMRRC) and on C57BL/6 genetic background. Mice were maintained in a 12 h light/dark cycle at 22–26 °C (lights on at 7:00 am) with ad libitum food and water. Animals were assigned randomly into different groups. Behavioral tests were conducted blindly.

### Stereotaxic injection

Virus was injected through a pulled glass micropipette fitted to 10 μL Hamilton syringe driven by a Stoelting *kd* scientific pump and guided by a micromanipulator (RWD, China). For the DRG injection, mouse (2–3 months) was anesthetized by isoflurane and L5 DRG was exposed by removal of the lateral processes of the vertebrae. H129-EGFP was injected to the depth of 0.3 mm from the surface of DRG at a 30° angle. The volume of each injection was 750 nL in a pumping rate of 75 nL/min.

For the spinal cord injection, mouse (2–4 months) was anesthetized by isoflurane and lumbar spinal column was exposed by incision of skin and dissection of muscles. Mouse was mounted on a custom spine stabilizer (RWD). Virus was injected into vertebrae innervated by L4 and L5 DRGs unilaterally. For the dorsal spinal cord, virus was injected to the depth of 0.7 mm from midline at a 45° angle. The volume of each injection was 300 nL in a pumping rate of 50 nL/min.

For the brain injection, mouse (2–4 months) was anesthetized with isoflurane followed by dexmedetomidine. The mouse was placed on a stereotaxic apparatus (RWD) and kept anesthetized by isoflurane. Ophthalmic ointment was used to maintain eye lubrication and protect mouse from light. The skull over the SSpll and MOp of both hemispheres was carefully removed. Injections were conducted to the target nuclei in a rate of 20 nL/min. The total injection volume was adapted to the size of targeted brain nuclei.

For the two-photon in vivo imaging, a circular craniotomy (diameter, 6–7 mm) was made over bilateral S1HL, followed by injection of AAV2/9-hSyn-flex-GCaMP6s. After injection, a 4-mm diameter glass coverslip was implanted at the craniotomy. Then, a titanium head-plate was attached to the skull and fixed by dental cement.

After injection, the mouse was placed on a heating blanket to recover from anesthesia before returning to the home cage. The following functional experiments were performed at least 4 weeks after virus injection.

H129-EGFP was purchased from Brain Case (Shenzhen, China). AAV2/1-hSyn-Cre (1.0 × 10^13^ v.g./mL), AAV2/retro-hSyn-Cre (5.0 × 10^12^ v.g./mL), AAV2/9-EF1a-DIO-ChR2-EYFP (4.0 × 10^12^ v.g./mL), AAV2/9-EF1a-DIO-NpHR3.0-EYFP (4.0 × 10^12^  v.g./mL), AAV2/9-EF1a-DIO-EYFP (2.0 × 10^12^ v.g./mL), AAV2/9-EF1a-DIO-hM3Dq-mCherry (3.0 × 10^12^ v.g./mL), AAV2/9-EF1a-DIO-hM4Di-mCherry (2.0 × 10^12^ v.g./mL), AAV2/9-EF1a-DIO-mCherry (2.0 × 10^12^ v.g./mL), AAV2/9-EF1a-hM3Dq-mCherry (4.0 × 10^12^ v.g./mL), AAV2/9-EF1a-hM4Di-mCherry (4.0 × 10^12^ v.g./mL), AAV2/9-EF1a-mCherry (3.0 × 10^12^ v.g./mL), AAV2/9-hSyn-mCherry (5.0 × 10^12^ v.g./mL), AAV2/9-hSyn-ChR2-mCherry (4.0 × 10^12^ v.g./mL), AAV2/9-hSyn-NpHR3.0-mCherry (5.0 × 10^12^ v.g./mL), AAV2/9-EF1a-dAPEX2 (5.0 × 10^12^ v.g./mL), AAV2/9-EF1a-DIO-COX4-dAPEX2 (4.0 × 10^12^ v.g./mL), EnvA-RV-ΔG-DsRed (5.0 × 10^8^ v.g./mL) and N2C-RV-ΔG-DsRed (3.0 × 10^8^ v.g./mL) were purchased from BrainVTA (Wuhan, China). AAV2/9-hSyn-flex-GCaMP6s (4.0 × 10^12^ v.g./mL), AAV2/9-CAG-DIO-EBFP (3.0 × 10^12^ v.g./mL) and AAV2/9-hSyn-EGFP (5.0 × 10^12^ v.g./mL) were purchased from Taitool Bioscience (Shanghai, China). AAV2/9-DIO-RVG-TVA-EGFP (5.0 × 10^12^  v.g./mL), slAAV (5.0 × 10^12^ v.g./mL), retargeted H129 recombinant Hs06-mCherry, AAV2/9-hSyn-DIO-EGFP-T2A-Her2CT9 and AAV2/9-UL26.5p-DIO-cmgD were constructed previously.

### Two-photon imaging in awake mouse, data analysis and quantification

The mouse strain was Scnn1a-Cre. To implant the cranial window, the mouse (2–4 months) was immobilized in custom-built stage-mounted ear bars and a nosepiece, similar to a stereotaxic apparatus. A 2-cm incision was made between the ears, and the scalp was stripped to expose the skull. One circular craniotomy (6–7 mm in diameter) was made using a high-speed drill, and a dissecting microscope was used for gross visualization. The skull was removed and 500 nL AAV2/9-hSyn-flex-GCaMP6s was pressure-injected at a depth of 400 μm under the surface of cerebral cortex. A glass coverslip (8 mm in diameter) was attached to the skull using dental cement. The space between the coverslip and cortical surface was filled with dulbecco phosphate buffered saline before sealing. The sterile technique was critical to prevent the infection of the bone. After a recovery period of 4 weeks from the surgery, the awake mouse was subjected to two-photon imaging. The mouse was in the head-fixed condition, and the body of mouse was constrained in a tube to prevent strong movements. No motion/movement of the brain is detected during imaging. For the electrical stimulation, 30 V constant voltage (50 Hz, 200 ms) was applied with the metal electrodes to the plantar surface of mouse hindpaws. The pain-related audible vocalization could be induced during the electrical stimulation. GCaMP6s fluorescence intensity in the cortical SSpll and MOp areas was imaged with an Olympus FVMPE-RS microscope coupled with a 2-mm working distance, 25× water immersion lens (numerical aperture, 1.05). A mode-locked InSight X3-OL femtosecond IR laser (Spectra-Physics) generated two-photon excitation at 960 nm for GCaMP6s fluorescence. The emission spectrum of GCaMP6s is 495–540 nm collected by a thermoelectric/air-cooled GaAsP photomultiplier tube (Hamamatsu). The power reaching the mouse brain was 7–15 mW. Images were acquired at 33.3 ms/frame at a resolution of 512 × 512 pixels.

The stack images were aligned with customized MatLab software (The MathWorks). The images from each session were first realigned to a reference image (the average image of all stable frames) using a normalized cross-correlation-based translation algorithm to correct the *x*-*y* offset of images to compensate for the motion between the objective and the cortex. Using ImageJ software (National Institutes of Health, USA), we manually identified the cell bodies and calculated the average fluorescence intensity of the regions of interest. The baseline of responsive neurons was calculated by averaging the calcium signals of 10 s before the beginning of each trial. The values of calcium transients change (ΔF/F) was aligned to individual trial baseline. The exact time that electrical stimulation begins was marked by the electrical input to the monitor. In the line scanning experiment, the response latency of neuron was defined as the interval between the time that the electric stimulus begins and the time that the calcium signal of monitored neurons reached 10% higher than the baseline. Data were analyzed with the custom-written code in MatLab, and presented as mean ± SEM.

### Two-photon imaging on brain slice

The mouse was deeply anesthetized, and then the brain was dissected out and placed in ice-cold ACSF. Then the acute brain slice preparations (400 μm thick) were made with a vibratome in ice-cold ACSF (oxygenated with 95% O_2_, 5% CO_2_). Given the low expression and fluorescence intensity of mCherry compared to that of GCaMP6s in the brain slice, the slice containing mCherry-positive spinal ascending axons and GCaMP6s-positive cortical descending axons in SCCD was examined immediately under a fluorescence microscope with emission filters ranging from 470 nm to 550 nm and from 567 nm to 643 nm to observe GCaMP6s and mCherry signals, respectively. Only one slice could be selected for in vitro calcium imaging because the thickness of SCCD was ~50 μm. Then the slice was embedded in the low-melting-point agarose and incubated in oxygenated ACSF containing TTX and 4-AP in a 35-mm dish. The embedded brain slice was imaged with an Olympus FVMPE-RS microscope coupled with a 2-mm working distance, 25× water immersion lens (numerical aperture, 1.05). A single mode-locked InSight X3-OL femtosecond IR laser (Spectra-Physics) generated two-photon excitation at 960 nm for GCaMP6s and at 1040 nm for mCherry. The emission spectra of GCaMP6s and mCherry are 495–540 nm and 575–645 nm, respectively. The 960 nm excitation wavelength could produce an emission efficiency of ~90% for GCaMP6s and only ~40% for mCherry. To avoid fluorescence bleaching of GCaMP6s, the power reaching the brain slice ranged from 7 mW to 10 mW. Therefore, the GCaMP6s and mCherry signals were recorded by 960 nm and 1040 nm wavelengths before CNO incubation, respectively. Only the change of GCaMP6s fluorescence intensity induced by presynaptic hM3Dq activation was monitored during the application of oxygenated ACSF containing CNO, TTX and 4-AP. The presynaptic spinal ascending fibers containing hM3Dq-mCherry were activated by CNO. The incubation with TTX and 4-AP was to prevent the polysynaptic transmission. The responsive calcium signals of GCaMP6s-positive cortical descending axons observed in SCCD during the experiment would be monosynaptically activated by presynaptic hM3Dq-containing spinal ascending terminals. To maintain the activity of embedded slice, the calcium activity of only single scanning layer (~1 μm) was monitored within 30 min.

### Behavioral tests

Behavioral tests were performed with 2–4-month-old male mice. Mouse was habituated in the experimental environment for at least 1 h per day for over 3 days before formal behavioral tests. In the Hargreaves and hot plate tests, the response latency to noxious heat stimuli was recorded. In von Frey test, the threshold of noxious mechanical response was measured. The above tests were stopped at a cutoff time or force for the animal protection.

### Hargreaves test

Mouse was habituated in a small (15 cm × 15 cm × 7.5 cm) plastic chamber on glass floor, and radiant light (Ugo Basile, 37300) was applied to left hindpaw of mouse. The light was applied when the mouse was resting quietly and was stopped after the quick movement of the hindpaw. The cutoff time was 20 s.

### Hot plate test

Mouse was put on a hot plate (Ugo Basile, 35150) at a temperature of 52 °C, and the cutoff time was 30 s.

### von Frey test

Each mouse was habituated in a small (15 cm × 15 cm × 7.5 cm) plastic chamber on a mesh floor for at least 30 min before testing. The mechanical threshold was measured with a series of von Frey filaments (Ugo Basile, 37450-275) applied to the mouse hindpaw. Each filament was applied 5 times (at least 10 s interval) in increasing order from the lowest force. The response to at least three of the five stimulations was determined as the mechanical response threshold.

To prepare the neuropathic pain model induced by SNI, we transected the common peroneal and tibial branches of the left sciatic nerve with ~0.5 cm of nerve removed and left the sural nerve intact. The von Frey test was performed on the lateral part of the left plantar surface where the sural nerve innervates.

### Foot shock escape test

The mouse was placed to the box, followed by 3 trials of electric foot shock (1 s, 0.5 mA constant current, at 1 min, 3 min, and 5 min, respectively). The movie was captured by a camera with the frequency of 30 frames/s. The first frame of mouse that began to escape was recorded as the escape latency of the test.

### Rotarod test

The mouse was tested on a rotarod with the velocity increasing from 5 rpm to 40 rpm within 5 min. The duration time on the rotarod before the mouse fell off was measured.

### Beam walking test

The mouse was placed on the training beam (28 mm diameter, square) and allowed to transverse the beam within 60 s. Four constitutive trials were performed and then the mouse was returned to the home cage. Three constitutive training days were required before the formal test.

On the test day, two constitutive trials were performed on each of square and round beams. The mouse was first tested with the widest beam and progress to the narrowest beam. The latency to transverse each beam was recorded and the cutoff time was 60 s.

### Footprint analysis

The forepaws and hindpaws were painted with red ink and black ink, respectively. Then, the mouse was put on the end of the paper sheet opposite to the goal box. The mouse was placed in a clean cage after running over the paper to the goal box. The parameters were measured from the footprint patterns.

### Open field test

The exploratory locomotor activity of mouse in a 30-min period was recorded in an open field (45 cm × 45 cm) apparatus. The total distance of horizontal moving during the whole procedure was measured.

### Tissue processing and imaging

Mouse was perfused transcardially with 0.9% saline followed by 4% paraformaldehyde in PBS. The DRG, spinal cord and brain were dissected, and then fixed overnight in 4% paraformaldehyde at 4 °C.

The brain was dehydrated in 30% sucrose solution at 4 °C until it sank to the bottom. Then, the brain was embedded in O.C.T compound (SAKURA), and a complete series of 40-μm coronal sections were gathered from each brain by Leica CM 1950. The DRG and spinal cord were dehydrated in 20% sucrose solution at 4 °C for at least 2 days. Then, the spinal cord was embedded in O.C.T compound, and 20-μm coronal sections of the lumbar spinal cord and 10-μm coronal sections of the DRG were collected by Leica CM1950.

Sections were mounted on slides and imaged with a confocal microscope (Leica SP8). The anatomical location and fluorescent EYFP, EGFP, mCherry, DsRed, and tdTomato in brain sections were matched to the structures according to nomenclature of the Allen Brain Atlas.

### Immunohistochemistry

For the brain, brain slices were blocked in PBS containing 5% bovine serum albumin and 0.3% Triton X-100 for 1 h at room temperature. The slices were incubated with chicken anti-GFP (Abcam, ab13970, 1:2000), rabbit anti-RFP (MBL, PM005, 1:2000), goat anti-mCherry (SICGEN, AB0040-20, 1:2000), rabbit anti-synapsin 1 (Chemicon, AB1543, 1:500) or/and rabbit anti-homer1 (Synaptic System, 160003, 1:500) antibodies for > 18 h at 4 °C, followed by incubation with corresponding secondary antibodies (Invitrogen) for 1 h at room temperature. The slices were finally mounted on glass slides and coverslipped with mounting medium.

For the DRG and spinal cord, segments were blocked in PBS containing 10% donkey serum, 0.1% gelatin and 0.05% Triton X-100 for 2 h at room temperature. The segments were incubated with chicken anti-GFP, rabbit anti-RFP or/and goat anti-mCherry antibodies for > 18 h at 4 °C, followed by incubation with corresponding secondary antibodies for 45 min at 37 °C. The slices were then coverslipped with mounting medium.

### Multiplexed peroxidase-based electron microscopy

The mouse following injection of AAV2/9-EF1a-dAPEX2 (Addgene, 117173) and AAV2/9-EF1a-DIO-COX4-dAPEX2 (Addgene, 117177) to express peroxidase dAPEX2 was perfused transcardially with 2% paraformaldehyde and 2.5% glutaraldehyde in cacodylate buffer (0.15 M sodium cacodylate and 0.04% CaCl_2_, pH 7.4) after Ames’ medium (oxygenated with 95% O_2_, 5% CO_2_, warmed to 37 °C). The brain was dissected and fixed in the same fixative buffer at 4 °C overnight. Then, the brain was cut by a vibratome (Leica) to 100 μm slices in ice-cold cacodylate buffer. The brain slices were incubated with 1 mL of DAB (0.3 mg/mL) in cacodylate buffer in the dark for 30 min, and 10 μL of 0.3% H_2_O_2_ was added into the buffer to start the peroxidase reaction. The reaction lasted 1 h with protection from light and then the brain slices were washed with cacodylate buffer. The peroxidase-positive area at the SCCD was selected by a fluorescent microscope, cut into 1 mm × 1 mm slice and fixed with cacodylate buffer containing 3% glutaraldehyde at 4 °C overnight. The slices were fixed in 1% osmium tetroxide for 1 h and then stained in a solution containing 1% uranyl acetate at 4 °C overnight. Then, the slices were dehydrated with increasing concentrations of ethanol, passed through propylene oxide, and embedded with Epon 812. Samples were sectioned using a Leica EM ultramicrotome. Ultrathin sections (70 nm) were examined by FEI Talos L120C TEM (Thermo Fisher Scientific).

### Implantation of optical fiber and optogenetic manipulation

To optically stimulate cell bodies, optic fibers (NA: 0.37; NEWDOON, Hangzhou, China) were bilaterally implanted into SSpll (AP, –0.85 mm; ML, ±1.35 mm; DV, –0.5 mm). To stimulate the terminals of projection neurons at the SCCD, an optic fiber was unilaterally implanted into SCCD (AP, –4.8 mm; ML, –0.2 mm; DV, –4.4 mm). Mouse would rest for at least 4 weeks to recover after the surgery. For the optogenetic ChR2 activation, mouse received 473 nm blue light laser (NEWDOON) with the power of 5–8 mW for soma stimulation or 15–20 mW for terminal stimulation at the fiber tip. For the optogenetic NpHR inhibition, mouse received 589 nm yellow light laser (Aurora-220-589, NEWDOON) with the power of 8–10 mW for soma stimulation or 15–20 mW for terminals stimulation at the fiber tip.

### BPN damage by optical fiber

To physically damage the BPN, optic fibers (NA: 0.37; NEWDOON) were bilaterally implanted into the BPN (AP, –4.8 mm; ML, –0.2 mm; DV, –4.75 mm). To avoid injury of the arteries on the dorsal surface of the brain, the fiber was angled 15° to the vertical. Mouse would rest for at least 4 weeks to recover from the surgery.

### DREADD activation and inhibition

CNO (Sigma-Aldrich, C0832) was prepared as a 1 mg/mL stock solution in DMSO, and then diluted in saline to a final concentration of 100 μg/mL. This solution was injected intraperitoneally into mouse to achieve final dose of 1 mg/kg. All behavioral experiments began at least 30 min after CNO injection.

### Experimental design and statistical analysis

Statistical analysis was performed using Prism 8 (GraphPad Software) and MATLAB 2013b (MathWorks). All data were obtained from at least 3 independent experiments, and presented as mean ± SEM. The data were analyzed using two-tailed *t*-test, two-way ANOVA test followed by Bonferroni correction or K–S test according to the experiments. All data met the assumption of the statistical tests used. Differences were regarded as significant at *P* < 0.05.

### Supplementary information

Supplementary information, Fig. S1

Supplementary information, Fig. S2

Supplementary information, Fig. S3

Supplementary information, Fig. S4

Supplementary information, Fig. S5

Supplementary information, Fig. S6

Supplementary information, Fig. S7

Supplementary information, Video S1

Supplementary information, Video S2

Supplementary information, Video S3

Supplementary video legend
