# Supplementary material for: A direct spino-cortical circuit bypassing the thalamus modulates nociception
Source: Cell Res. 2023 Jun 13;33(10):775–89. doi: 10.1038/s41422-023-00832-0 (PMC10542357; doi:10.1038/s41422-023-00832-0)
Supplement: Supplementary file 1 — Supplementary information, Fig. S1 [file 41422_2023_832_MOESM1_ESM.pdf]

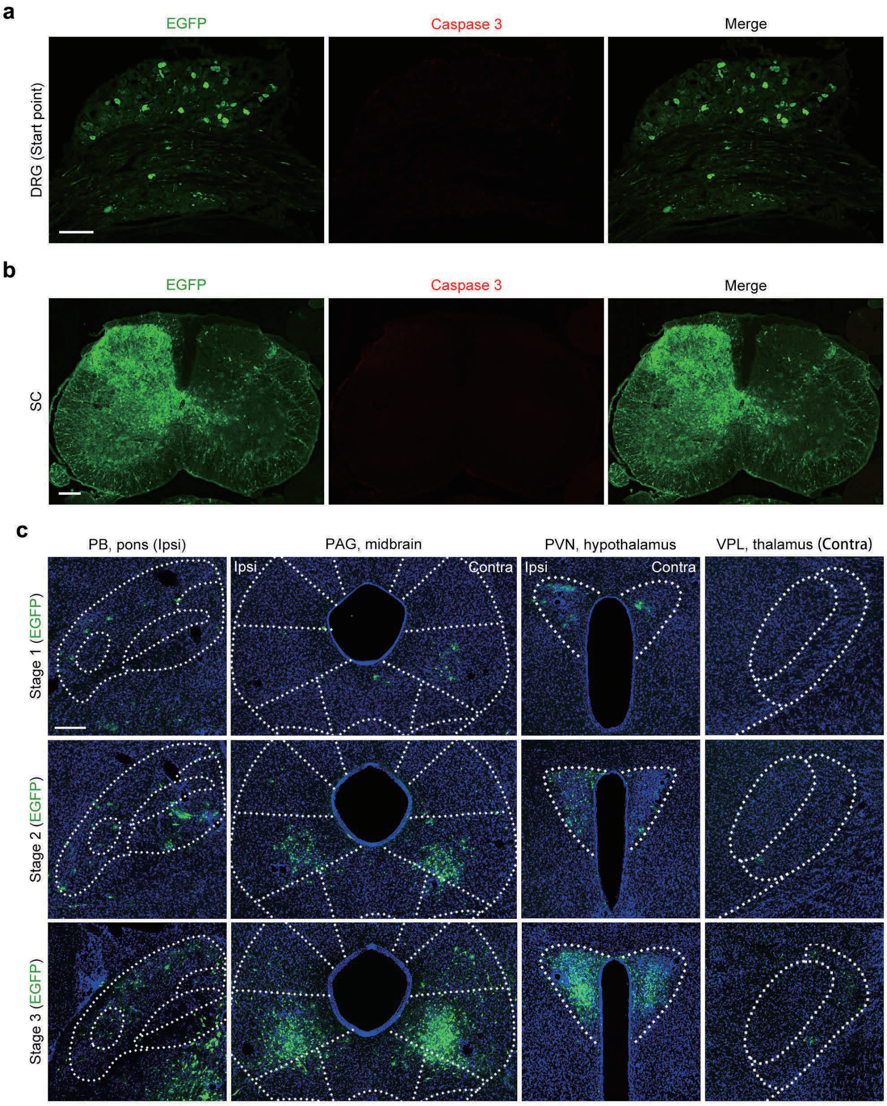

Supplementary information Fig. S1 Tracing of the somatosensory neural network in the brain arising from lumbar 5 DRG neurons. a, The expanded images of DRG in Fig. 1c showing EGFP-labeled DRG neurons did not express Caspase 3 (n = 19). Scale bar, 200 μm. **b,** The expanded images of SC in Fig. 1c showing EGFP-labeled neurons in the SC did not express Caspase 3 (n = 19). Scale bar, 200 μm. **c**, EGFP-labeled neurons in the ipsilateral PB, PAG, PVN and contralateral VPL. Scale bar, 200 μm (n = 8 for stage 1, n = 5 for stage 2, n = 6 for stage 3).
