# Supplementary material for: A direct spino-cortical circuit bypassing the thalamus modulates nociception
Source: Cell Res. 2023 Jun 13;33(10):775–89. doi: 10.1038/s41422-023-00832-0 (PMC10542357; doi:10.1038/s41422-023-00832-0)
Supplement: Supplementary file 2 — Supplementary information, Fig. S2 [file 41422_2023_832_MOESM2_ESM.pdf]

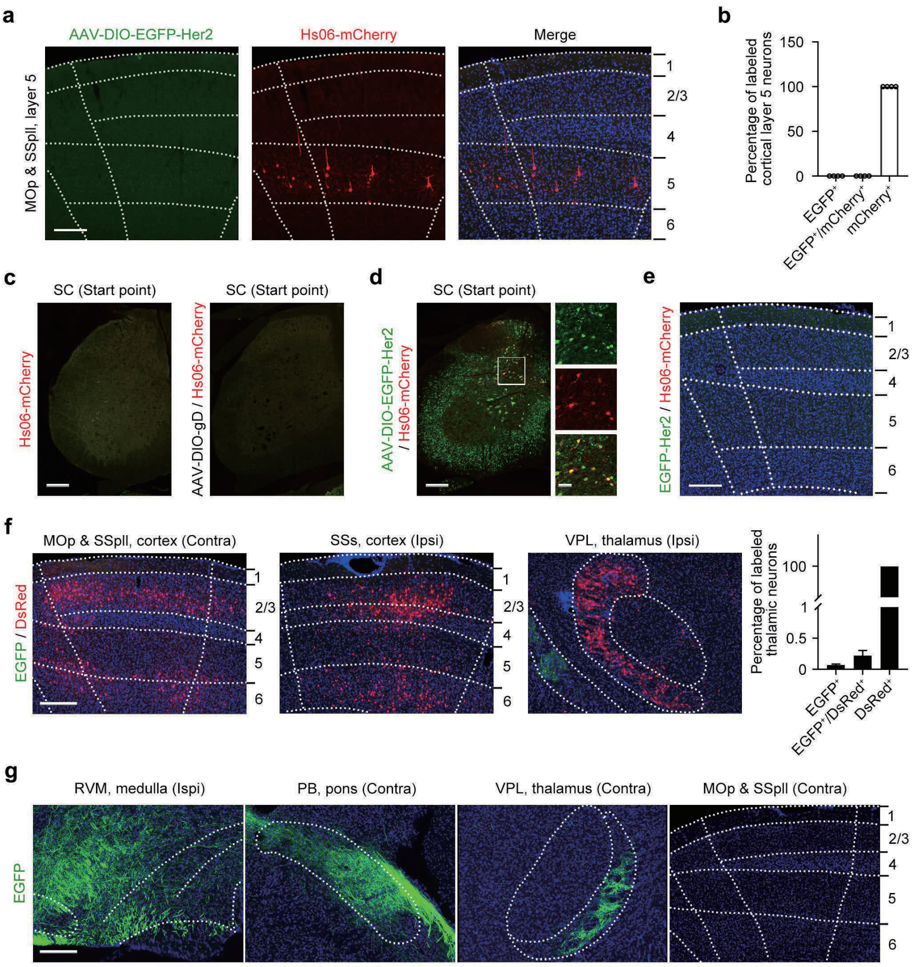

Supplementary information Fig. S2 Verifying the direct projection from lumbar SC to cerebral cortex by anterogradely and retrogradely trans-monosynaptic tracing. a, The expanded images of Fig. 1h showing only mCherry-labeled layer 5 neurons in the MOp and SSpll (n = 4). Scale bar, 200 µm. **b,** Statistical result showing 100% of labeled neurons in cortical layer 5 only expressed mCherry (n = 4). **c,** Representative images showing no mCherry-expressing neurons in the SC without the compensation of Her2 (n = 3 for both groups). Scale bar, 200 µm. **d,** Representative image showing the infection of Hs06 of Her2-expressing neurons in the SC (n = 3). Left scale bar, 200 µm, right scale bar, 50 µm. **e,** Representative image showing no

mCherry-labeled or EGFP-labeled neurons in SSpll and MOp of mice only expressing EGFP-Her2 in the SC (n = 3). Scale bar, 200 μm. **f,** Left, DsRed-labeled neurons in the contralateral SSpll, ipsilateral SSs and the ipsilateral VPL. Right, statistical result showing only ~0.2% DsRed-labeled thalamic neurons expressed EGFP (n = 3). Scale bar, 200 μm. **g,** EGFP-labeled axon fibers were observed in the ipsilateral RVM, the contralateral PB and the contralateral VPL but not in the contralateral sensorimotor cortex (n = 3). Scale bar, 200 μm. Data shown are mean ± S.E.M.
