# Supplementary material for: A direct spino-cortical circuit bypassing the thalamus modulates nociception
Source: Cell Res. 2023 Jun 13;33(10):775–89. doi: 10.1038/s41422-023-00832-0 (PMC10542357; doi:10.1038/s41422-023-00832-0)
Supplement: Supplementary file 3 — Supplementary information, Fig. S3 [file 41422_2023_832_MOESM3_ESM.pdf]

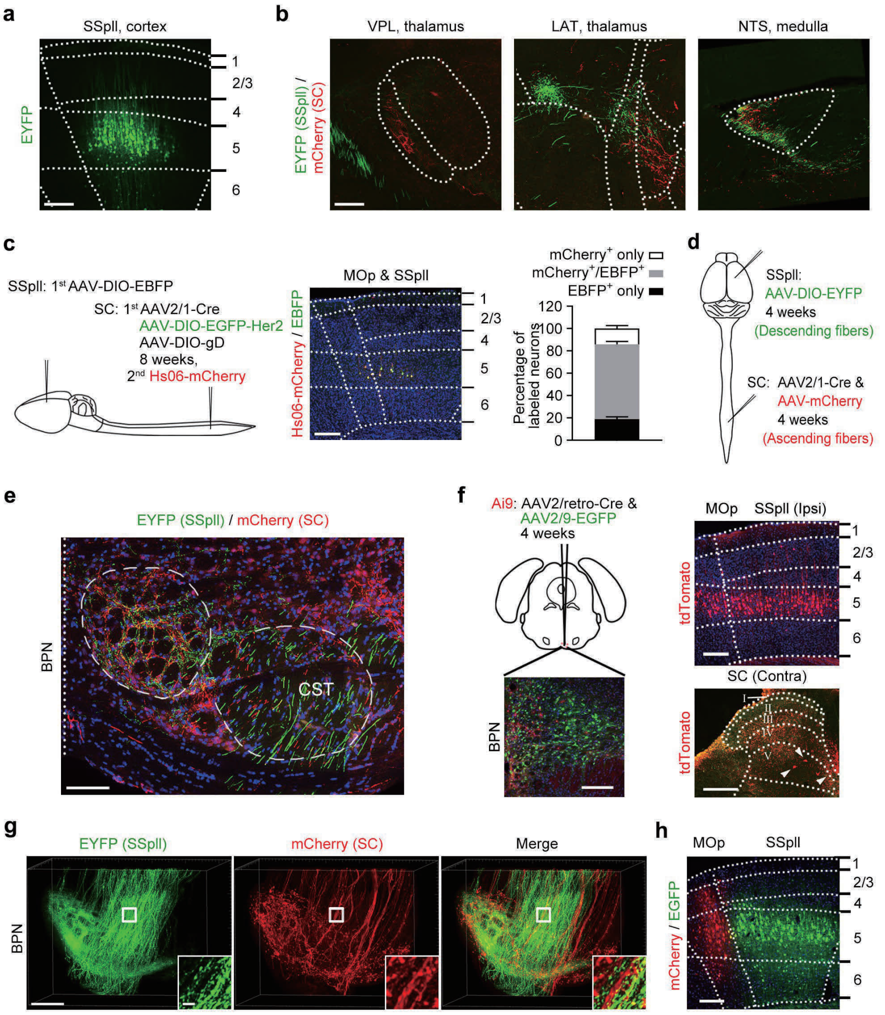

Supplementary information Fig. S3 Brain nuclei co-innervated by the axons from layer 5 neurons of SSpll and SPNs. a, A representative image showing EYFP-labeled neurons in layer 5 of SSpll in Rbp4-Cre mice (n = 3). Scale bar, 200 μm. **b,** EYFP-labeled axons from layer 5 neurons of SSpll and mCherry-labeled axons from SPNs in VPL, LAT, and NTS (n = 3). Scale bar, 200 μm. **c,** Schematic, representative image and statistical result showing viral injections to label SCRNs by both Hs06 and AAV2/1-Cre (n = 4). Scale bar, 200 μm. **d,** Schematic showing viral injections to express mCherry in SPNs and EYFP in SCRNs of wild-type mice. **e,** The distribution of EYFP-

labeled axon fibers from SCRNs and mCherry-labeled axon fibers from SPNs in the BPN of wild-type mice (n = 3). Scale bar, 100 µm. The vertical dotted line indicates the midline of the coronal section in the brain. **f,** Schematic (top left) and the image (bottom left) showing the injection of AAVs into the BPN of Ai9 mice. Top right, tdTomato-labeled neurons in layer 5 of SSpll and MOp. Bottom right, tdTomato-labeled neurons in deep laminae of contralateral lumbar spinal cord (n = 3). Scale bars, 200 µm. **g,** The expanded images of Fig .2f showing the SCCD across CST in the BPN formed by EYFP-labeled axon fibers from SCRNs and mCherry-labeled axon fibers from SPNs (n = 4). The image was captured from the 10th sec of supplementary information video S1. Scale bar, 200 µm. The enlarged images were captured from the 1st sec of supplementary information video S1 (anticlockwise 16° to the original images). Scale bar, 50 µm. **h,** Expression of mCherry and EGFP in MOp and SSpll, respectively (n = 3). Scale bar, 200 µm.
