# Supplementary material for: A direct spino-cortical circuit bypassing the thalamus modulates nociception
Source: Cell Res. 2023 Jun 13;33(10):775–89. doi: 10.1038/s41422-023-00832-0 (PMC10542357; doi:10.1038/s41422-023-00832-0)
Supplement: Supplementary file 4 — Supplementary information, Fig. S4 [file 41422_2023_832_MOESM4_ESM.pdf]

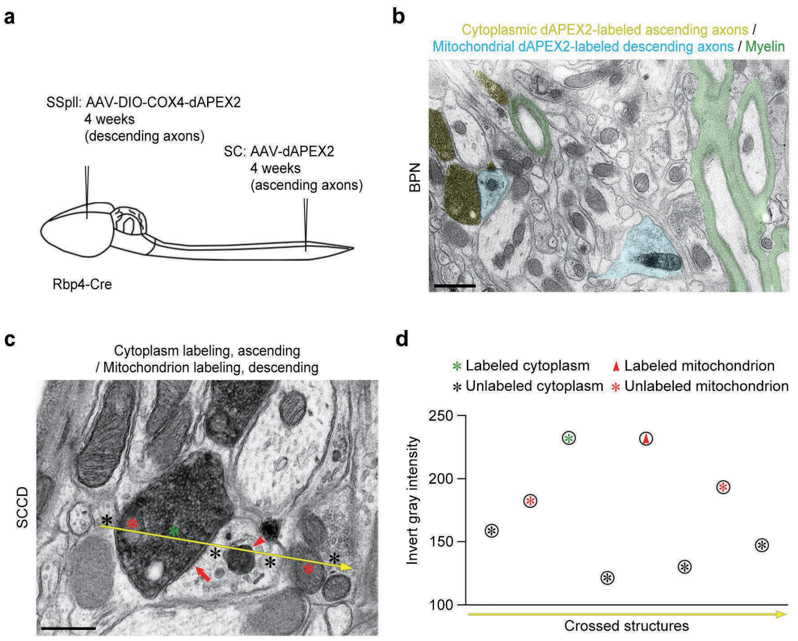

Supplementary information Fig. S4 Functional synaptic contacts at axon terminals from SPNs and SCRNs in the BPN. a, Schematic showing injections of cytoplasmic peroxidase-expressing AAV into spinal cord and mitochondrial peroxidase-expressing AAV into SSpll in Rbp4-Cre mice. **b,** The expanded image of electron microscopy showing the synapse between labeled axon terminals from SPNs (cytoplasmic labeled peroxidase, green asterisk, yellow mask) and SCRNs (mitochondrial labeled peroxidase, black asterisk, blue mask) in the BPN. The green masks indicate myelin in the BPN (n = 3). Scale bar, 1 μm **c,** The expanded image of Fig. 2i showing more unlabeled structures around the labeled synapse (n = 3). The black asterisks indicate unlabeled cytoplasm and red asterisks indicate unlabeled mitochondria. The yellow line with arrow crosses eight different labeled or unlabeled subcellular structures. Scale bar, 500 nm. **d,** Invert gray intensity of crossed structures by the yellow line with arrow.
