# Supplementary material for: A direct spino-cortical circuit bypassing the thalamus modulates nociception
Source: Cell Res. 2023 Jun 13;33(10):775–89. doi: 10.1038/s41422-023-00832-0 (PMC10542357; doi:10.1038/s41422-023-00832-0)
Supplement: Supplementary file 5 — Supplementary information, Fig. S5 [file 41422_2023_832_MOESM5_ESM.pdf]

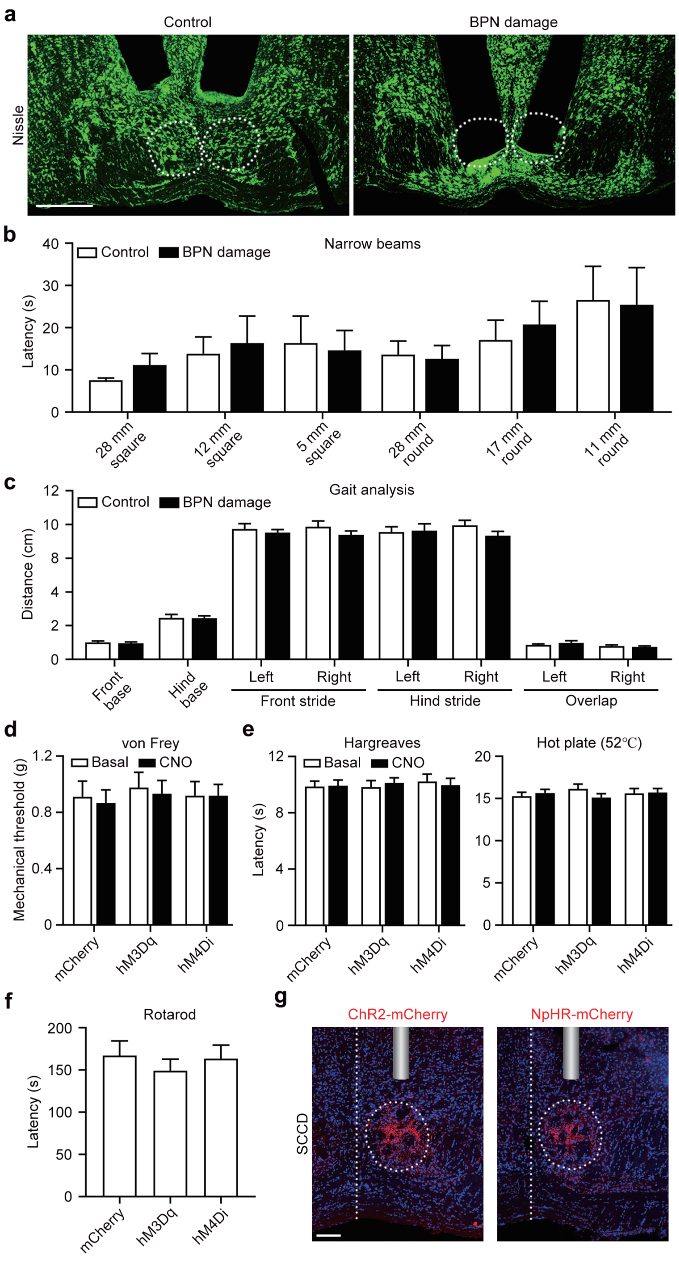

Supplementary information Fig. S5 Motor ability after BPN damage. a, Representative images showing control and BPN damage with ceramic fibers. Scale bar, 200 μm. **b,** The latencies to transverse beams after BPN damage. n = 7 for control group and n = 6 for BPN-damaged group. **c,** The gait analysis of BPN-damaged mice. n = 7 for control group and n = 6 for BPN-damaged group. **d,** Nociceptive mechanical threshold in von Frey test after chemgoenetic manipulation of BPN local neurons. n =

9 for both mCherry and hM3Dq groups, n = 10 for hM4Di group. **e,** Noxious thermal latencies in hargreaves test and hot plate test after chemgoenetic manipulation of BPN local neurons. n = 9 for both mCherry and hM3Dq groups, n = 10 for hM4Di group. **f,** Rotarod test showing the motor ability after chemgoenetic manipulation of BPN local neurons. n = 9 for both mCherry and hM3Dq groups, n = 10 for hM4Di group. **g,** Axon fibers from SPNs expressing ChR2-mCherry (left) or NpHR-mCherry (right) at the SCCD. Scale bar, 100 μm. Data shown are mean ± S.E.M. Two-tailed unpaired *t*-test.
