# Supplementary material for: A direct spino-cortical circuit bypassing the thalamus modulates nociception
Source: Cell Res. 2023 Jun 13;33(10):775–89. doi: 10.1038/s41422-023-00832-0 (PMC10542357; doi:10.1038/s41422-023-00832-0)
Supplement: Supplementary file 6 — Supplementary information, Fig. S6 [file 41422_2023_832_MOESM6_ESM.pdf]

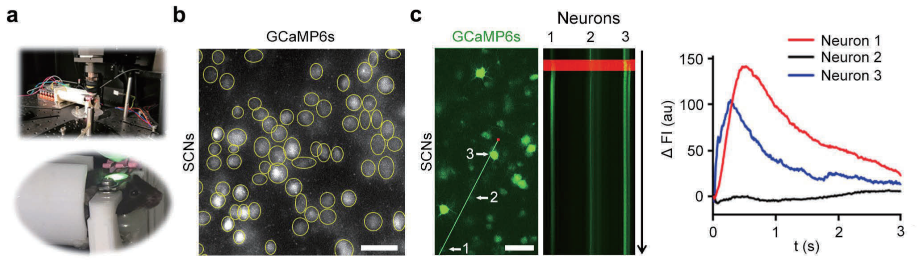

Supplementary information Fig. S6 Measurements of neuronal response latency via line scanning during *in vivo* calcium imaging. a, Images showing *in vivo* two photon calcium imaging performed on head-fixed awake mice. **b,** A representative image of GCaMP6s-labeled SCRNs in cortical layer 5 during the experiment (n = 3). Scale bar, 50 μm. **c,** Process of line scanning performed on single neuron. Left, a representative image showing GCaMP6s-labeled SCRNs via frame scanning. The line across the neurons indicates scanning solely between both ends of the line. Scale bar, 100 μm. Middle, a representative image showing that 3 neurons are contained in the line scanning. The red bar indicates the beginning of peripheral noxious electrical stimulation. Right, the response traces of 3 neurons during peripheral noxious electrical stimulation (n = 3).
