# Supplementary material for: A direct spino-cortical circuit bypassing the thalamus modulates nociception
Source: Cell Res. 2023 Jun 13;33(10):775–89. doi: 10.1038/s41422-023-00832-0 (PMC10542357; doi:10.1038/s41422-023-00832-0)
Supplement: Supplementary file 7 — Supplementary information, Fig. S7 [file 41422_2023_832_MOESM7_ESM.pdf]

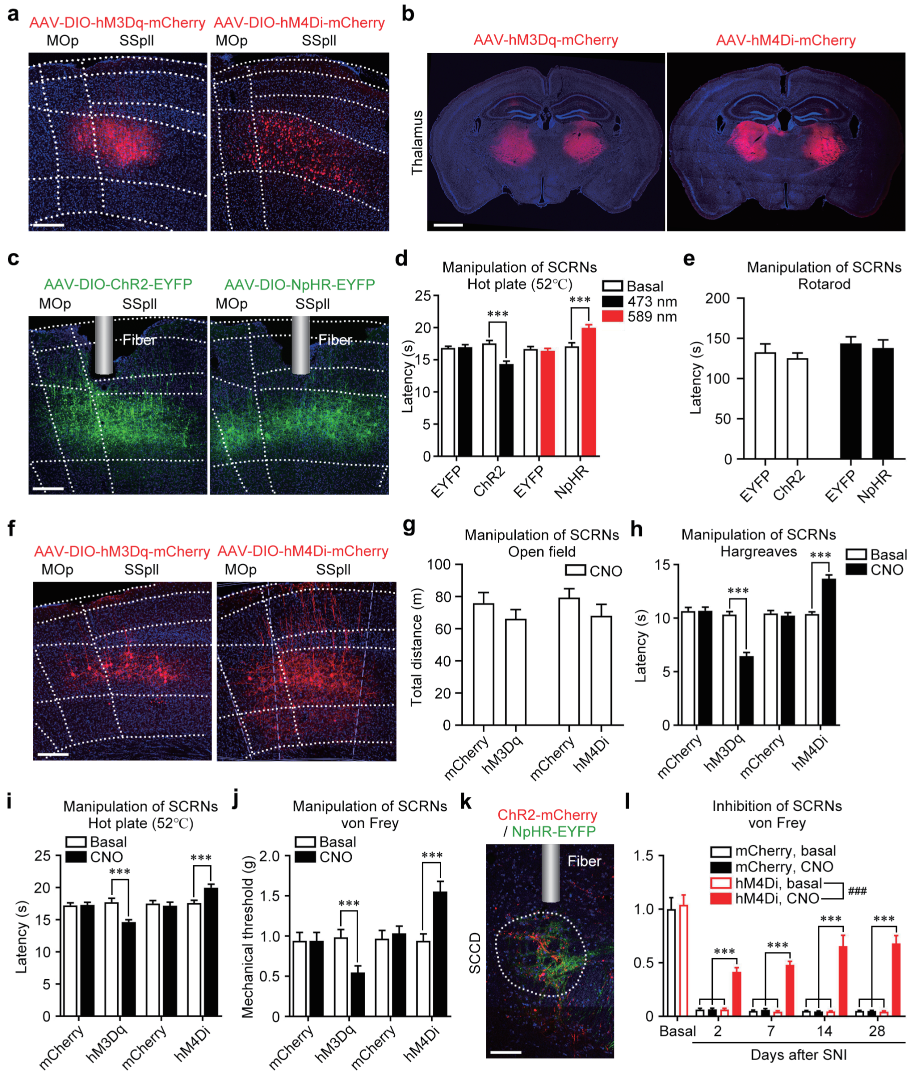

Supplementary information Fig. S7 Contribution of SCRNs to nociceptive responses. a, Representative images showing the expression of hM3Dq-mCherry or hM4Di-mCherry in layer 5 neurons of SSpll and MOp after the viral infection in Rbp4-Cre mice. Scale bar, 200 μm. **b,** Representative images showing the expression of hM3Dq-mCherry or hM4Di-mCherry in thalamic neurons in wild-type mice. Scale bar, 1 mm. **c,** Representative images showing the expression of ChR2-EYFP or NpHR-EYFP in SCRNs of SSpll and MOp after the viral infection. Scale bar, 200 μm. **d,** Noxious thermal latency in hot plate test after optogenetic activation and inhibition of

SCRNs. *** $p < 0.001$ v.s. basal, n = 12 for ChR2, n = 15 for EYFP, n = 14 for NpHR and n = 12 for EYFP groups. **e,** Rotarod test showing motor ability after optogenetic manipulations of SCRNs. n = 11 for ChR2, n = 10 for EYFP, n = 13 for NpHR and n = 14 for EYFP groups. **f,** Representative images showing the expression of hM3Dq-mCherry or hM4Di-mCherry in SCRNs in wild-type mice. Scale bar, 200 μm. **g,** Open field test showing the motor ability after chemogenetic manipulation of SCRNs. n = 13 for both hM3Dq and mCherry groups, and n = 14 for both hM4Di and mCherry groups. **h-j,** Noxious thermal latency in hargreaves test (h) and hotplate test (i), and nociceptive mechanical threshold in von Frey test (j) during chemogenetic manipulation of SCRNs. *** $p < 0.001$ v.s. basal. n = 12 (h, i) and n = 13 (j) for hM3Dq, n = 12 (h) and n = 13 (i, j) for mCherry groups, n = 11 (h) and n = 13 (i, j) for hM4Di, and n = 13 (h, i) and n = 12 (j) for mCherry groups. **k,** Image showing axon fibers from ChR2-mCherry-labeled SPNs and NpHR-EYFP-labeled SCRNs at the SCCD. Scale bar, 100 μm. **l,** Von Frey test after SNI showing the nociceptive mechanical threshold after chemogenetic inhibition of SCRNs. *** $p < 0.001$ v.s. controls, $^{\#\#\#}$ $p < 0.001$ v.s. hM4Di, basal. n = 11 for mCherry and n = 10 for hM4Di groups. Data shown are mean ± S.E.M. Two-tailed unpaired *t*-test (d, e, g-j) or two-way ANOVA test followed by Bonferroni correction (l).
