# Supplementary material for: A direct spino-cortical circuit bypassing the thalamus modulates nociception
Source: Cell Res. 2023 Jun 13;33(10):775–89. doi: 10.1038/s41422-023-00832-0 (PMC10542357; doi:10.1038/s41422-023-00832-0)
Supplement: Supplementary file 11 — Supplementary video legend [file 41422_2023_832_MOESM11_ESM.pdf]

**Supplementary information, Video S1** fMOST 3D reconstruction of the BPN.

**Supplementary information, Video S2** Layer 4 neuron responses.

**Supplementary information, Video S3** Layer 5 neuron responses.
